# Risks Associated With Benzodiazepine Long‐Term Use in Chronic Insomnia: A Systematic Review and (Network) Meta‐Analysis

**DOI:** 10.1111/jsr.70352

**Published:** 2026-06-11

**Authors:** Dieter Riemann, Chiara Baglioni, Christoph Nissen, Agathe Nevière, Omar Irfan, Pauline Le Nouveau, Chrysoula Leontiou, Damien Léger

**Affiliations:** ^1^ Department of Psychiatry and Psychotherapy, Medical Center University of Freiburg, Faculty of Medicine University of Freiburg Freiburg Germany; ^2^ Department of Human Sciences University of Rome 'G. Marconi'‐Telematic Rome Italy; ^3^ Department of Psychiatry University Hospital Geneva Geneva Switzerland; ^4^ Health Economics and Market Access Amaris Consulting Saint‐Herblain France; ^5^ Health Economics and Market Access Amaris Consulting Toronto Ontario Canada; ^6^ Idorsia Pharmaceuticals Ltd Allschwil Switzerland; ^7^ Université Paris Cité, VIFASOM (IMR Vigilance Fatigue et Santé Publique) Paris France

**Keywords:** benzodiazepines, chronic insomnia, insomnia, meta‐analysis, systematic review

## Abstract

This systematic literature review evaluated the risks of using benzodiazepine medications for more than 3 months in adults with chronic insomnia. Chronic insomnia is defined as ongoing dissatisfaction with sleep quantity or quality, causing significant distress and impaired functioning during the day. Although benzodiazepine medications are often prescribed to treat insomnia, using them longer than 4 weeks raises concerns about dependence, negative side effects, and long‐term health risks for patients. This review included 27 studies published between 1987 and 2023, focusing on adults with insomnia symptoms lasting at least 3 months. The main outcomes examined were sleep quality, safety issues such as risk of falls, mental health concerns including depression and anxiety, and overall quality of life. Five outcomes were deemed suitable for meta‐analysis: severity of insomnia, sleep quality, incidence of falls, depression, and anxiety. The results showed that people taking benzodiazepine medications had more severe insomnia than healthy sleepers, but less severe than those who were not taking any medication. The risk of falls was similar between those using benzodiazepine medications and those who were not. However, people using these medications experienced significantly higher levels of depression and anxiety compared to healthy sleepers. This review highlights a lack of studies on the long‐term use of benzodiazepine medications, but suggests that extended use may worsen sleep quality, mental health, and quality of life. The findings support guidelines recommending limited use and prioritizing behavioural therapy and alternative safer pharmacotherapies for insomnia.

## 1 Introduction

Insomnia is defined by the Diagnostic and Statistical Manual of Mental Disorders, Fifth Edition (DSM‐5) as a predominant dissatisfaction with sleep quantity or quality that results in significant distress and impaired daytime functioning (American Psychiatric Association 2023). All classifications relevant to sleep disorders, such as DSM‐5, the International Classification of Sleep Disorders, Third Edition‐TR (ICSD‐3TR), and more recently, the International Statistical Classification of Diseases and Related Health Problems, 11th Revision (ICD‐11), distinguish between acute and chronic insomnia, with a threshold of 3 months beyond which the condition is considered chronic (AMA 2023; Hafner et al. 2023; van Straten et al. 2025; Vargas et al. 2020) Population prevalence of chronic insomnia is reported to range from 6.0% to 14.8% (Hafner et al. 2023; van Straten et al. 2025), with an incidence rate of 8% (Hafner et al. 2023), and preferentially affects women and older adults (Vargas et al. 2020; Baglioni et al. 2020; Baldi et al. 2025). Insomnia adversely affects daytime and overall patient well‐being (Bollu and Kaur 2019; Grandner et al. 2023; Rosekind and Gregory 2010; Spira et al. 2014; Riemann et al. 2023; Huang et al. 2021). Chronic insomnia is associated with an increased risk of cardiovascular and mental disorders such as depression and anxiety disorders, as well as other neurological conditions compared to people without insomnia (Bollu and Kaur 2019; Grandner et al. 2023; Rosekind and Gregory 2010; Riemann et al. 2023; Li et al. 2016; Li et al. 2014; Fernandez‐Mendoza 2019; Havens et al. 2017; Brumpton et al. 2017; Benz et al. 2023; Hertenstein et al. 2023; Hertenstein et al. 2019; Baglioni et al. 2011). As a result, patients with insomnia have higher healthcare costs, absenteeism from school and work, and an increased risk of work and road accidents compared to the general population (Grandner et al. 2023; Riemann et al. 2023; Ellis et al. 2023; Al‐Shawwa et al. 2021; Espie et al. 2018; Morlock and Dobrescu 2019; Tzuang et al. 2021; Wickwire et al. 2016).

Cognitive behavioural therapy for insomnia (CBT‐I) is the recommended first‐line treatment for chronic insomnia disorder (Riemann et al. 2023; Hauk 2017; Sateia et al. 2017). If CBT‐I is ineffective, pharmacological interventions such as benzodiazepine receptor agonists (e.g., benzodiazepines (BZDs) or Z‐drugs (zolpidem, zopiclone, etc.)), or sedating antidepressants (off‐label use) can be prescribed for a short duration of up to 4 weeks (Palagini et al. 2023; Edinger et al. 2021; Zee et al. 2023). When longer‐term treatment is required, prolonged release melatonin could be considered for adults 55 years of age or older and most frequently for those with a suspected circadian rhythm disorder (Riemann et al. 2023). Dual orexin receptor antagonists (DORAs, e.g., daridorexant) have also shown effectiveness for up to 1 year (Kunz et al. 2023). However, despite benzodiazepine receptor agonists being considered for short term use, in clinical practice, use often spans longer periods of time (Zee et al. 2023; Lugoboni and Quaglio 2014; Fang et al. 2009; Kurko et al. 2015). A recent survey of the treatment landscape of chronic insomnia in five European countries (France, Germany, Italy, Spain, and UK) found the average duration of BZD use to be 15.3 weeks (Soyka et al. 2023), with additional studies reporting similar or higher rates of long‐term BZD use in other regions (Airagnes et al. 2019; Urru et al. 2015).

Ongoing concerns regarding long‐term use of BZDs and other hypnotic medications include increased risk for serious adverse events and long‐term outcomes, such as cognitive (particularly memory) impairment, substance abuse and tolerance, dependency, daytime sedation, and increased risk of falls and other accidents, especially among older adults (Pollmann et al. 2015; Uzun et al. 2010; Pottie et al. 2018). While previous systematic reviews have described the risks of BZD use, reviews of long‐term use of BZDs in patients with chronic insomnia and associated consequences are further limited (Buscemi et al. 2007; Rios et al. 2019; Nader and Gowing 2020). Therefore, it is important to further evaluate and understand the implications of long‐term use of hypnotic medications, particularly BZDs, among patients with chronic insomnia. This systematic literature review (SLR) aimed to identify risks associated with longer‐term (≥ 3 months) BZD use in patients with chronic insomnia, including outcomes related to sleep quality, safety concerns (e.g., alcohol abuse, falls risk, cognitive impairment), mental health issues (e.g., depression, anxiety), and effects on quality of life (QoL) (Kurko et al. 2015). An additional aim was to conduct a series of meta‐analyses to compare these outcomes in patients with chronic insomnia taking BZDs to patients not taking any medication for chronic insomnia and to healthy controls (i.e., good sleepers).

## 2 Methods

The protocol of this SLR has been registered with PROSPERO (CRD42022375623).

### 2.1 Search Strategy

A comprehensive search was conducted and reported according to Preferred Reporting Items for Systematic Reviews and Meta‐Analyses extension for reporting literature searches in systematic reviews (PRISMA‐S) and European Network for Health Technology Assessment (EUnetHTA) guidelines (Rethlefsen et al. 2021; Eunetha 2019).

The search strategy was executed in two phases: the publication date for studies of interest was restricted to 2012–2023 in the first phase of the SLR considering the implementation of the DSM‐5 definition of chronic insomnia but revealed a lack of relevant evidence; resulting in the search being further expanded to cover 1987–2011 in the second phase after reevaluation of the definitions, considered as not drastically different in the context of this study. The year 1987 denotes the publication of the Diagnostic and Statistical Manual of Mental Disorders, Third Edition, Revised (DSM–III‐R) criteria (American Psychiatric Association 1987) by the American Psychiatric Association due to observed inconsistencies and unclarity in the diagnostic system of the earlier DSM–III criteria (American Psychiatric Association 1980).

Structured searches of electronic databases were conducted between November 21, 2022 and January 4, 2023 for the original SLR timeframe, and between January 18, 2023 and February 2, 2023 for the extended timeframe. Grey literature searches, including conference proceedings published after 2012 and online repositories, were conducted on November 11, 2022 and February 2, 2023 for the original and extended SLRs, respectively. The electronic databases search included Embase, MEDLINE, PubMed, the Cochrane Central Register of Controlled Trials (CENTRAL), PsycNET covering PsycArticles and PsycInfo, and clinical trial registries (i.e., ClinicalTrials.gov and EU Clinical Trials Register). The search terms, containing MeSH/EMTREE terms and free text searches, used in the electronic database searches included chronic insomnia, insomnia, hyposomnia, sleep disorder, and any terms related to study design, and are presented in Supporting Information Tables 1–8, separated by database and date of search.

Sources for grey literature searches included the World Sleep Congress, the annual SLEEP meetings of the Associated Professional Sleep Societies (APSS), the European Sleep Research Society (ESRS) congress, the American Geriatric Society (AGS) congress, the European Union Geriatric Medicine Society (EUGMS) congress, the European Association for Palliative Care (EAPC) congress, the European Academy of Neurology (EAN) congress, World Organization of National Colleges, Academies and Academic Associations (WONCA), the US Food and Drug Administration (FDA) and European Medicines Agency (EMA) websites, surveys and reports from the Substance Abuse and Mental Health Services (SAMHSA), Drug Safety Research Unit (DSRU), and Google Scholar. Bibliographies of relevant systematic reviews and included studies were screened.

### 2.2 Study Selection

Adult patients (at least 18 years old) with a diagnosis of chronic insomnia were the population of interest. For inclusion of studies, different criteria for chronic insomnia were used depending on the date of publications: Diagnostic and Statistical Manual of Mental Disorders, Fourth Edition (DSM‐IV) criteria with a cutoff of at least 1 month of symptoms duration and National Institute of Sleep and Vigilance (INSV) (L'insomnie [Internet], n.d.), ICSD‐3 (Sateia 2014), and DSM‐5 (American Psychiatric Association 2013) criteria with at least 3 months of symptoms duration. Publications referring to insomnia due to other medical or mental health conditions were also considered. The intervention of interest was BZDs, with the duration of intervention (i.e., BZD use) being a minimum of 3 months for inclusion in the SLR. This cutoff for BZD use duration was selected to capture individuals using BZD for longer than the recommended treatment length of 4 weeks while including the longer 3‐month symptoms duration per the aforementioned diagnostic criteria. The included studies compared BZD use to an unrestricted list of comparators which included non‐BZD drugs, no intervention while having a diagnosis of chronic insomnia, waiting lists for BZD intervention, placebo, psychological interventions, and healthy controls (i.e., good sleepers). The broad categories for outcomes of interest included sleep quality, safety, mental health, and QoL outcomes (see Table 1 for examples of included outcomes). Both randomized and nonrandomized study designs were included, as well as observational studies, in order to capture comparisons between treatment and non‐treatment groups while reviews, case series, and case reports were excluded. Only publications in English language were considered for inclusion due to lack of translation resources. Further inclusion/exclusion criteria are as specified in Table 1.

**TABLE 1:** PICOS table outlining inclusion and exclusion criteria for the SLR search.

Category	Inclusion criteria	Exclusion criteria
Population	Adults (≥ 18 years of age) with chronic insomnia	Populations other than patients with chronic insomnia, Patients with chronic insomnia < 18 years of age
Intervention	BZD use > 3 months duration (outcome data after 3 months of treatment was used for long‐term safety)	Studies not reporting the use of BZD Studies reported a combination of treatments
Comparator	No restriction	Not applicable
Outcomes	Including but not restricted to: **Sleep Quality** ○ ISI ○ SII ○ PSQI ○ SSS ○ GSS ○ ESS ○ VAS **Safety** ○ Dependence ○ Abuse ○ Addiction ○ Tolerance ○ Cognitive impairment ○ Memory impairment ○ Dementia ○ Falls ○ Fatigue ○ Fractures, including hip fractures ○ Accidents: occupational and traffic accidents including near miss accidents and errors ○ Loss of productivity including absenteeism, loss of work, unemployment, and decreased caregiver role ○ Efficacy outcomes which can inform comparability of population during the feasibility assessment for the indirect treatment comparison **Mental Health** ○ Depression ○ Anxiety ○ Numbness ○ Irritability **QoL** ○ Quality‐adjusted life years ○ Loss of productivity including absenteeism, loss of work, unemployment, and decreased caregiver role	Any other outcomes or only efficacy outcomes
Study type	Observational studies, RCTs, nonRCTs, study registrations, study protocols	Reviews, case reports, case series
Publication type	Peer‐reviewed published articles, pre‐print articles, clinical trial registry entries, conference abstracts	Notes/editorials/letters, newspaper articles
Language	English	Non‐English
Publication year	January 1, 1987–present	Pre‐1987

Abbreviations: BZD, benzodiazepine; ESS, Epworth Sleepiness Scale; GSS, Groningen Sleep Scales; ISI, Insomnia Severity Index; nRCT, nonrandomized controlled trials; PICOS, population, intervention, comparator, outcome, study design; PSQI, Pittsburgh Sleep Quality Index; QoL, quality of life; RCT, randomized controlled trials; SII, Sleep Impairment Index; SLR, Systematic Literature Review; SSS, Stanford Sleepiness Scale; VAS, Visual Analogue Scale.

The selection of studies retrieved by the two phases of the search was conducted separately as per the inclusion/exclusion criteria specified in Table 1. These criteria were used on the de‐duplicated titles and abstracts identified by the search strategy when screened independently by two reviewers (Q.L.K., L.D.) for inclusion. Full texts of the included articles were further retrieved and reviewed for inclusion. Any discrepancies in screening and assessment were resolved by discussion to reach a consensus.

### 2.3 Data Extraction

Data extraction was performed using a standardized data extraction template. The data items extracted were details of publication, study design, population, intervention, comparators, and outcomes. Two reviewers (Q.L.K., L.D.) extracted the data, and a third reviewer (O.I.) quality‐checked the data extracted for any missing information or discrepancies. Any disagreements were resolved through discussion involving the three reviewers.

### 2.4 Risk of Bias Assessment

Risk of bias assessment was performed (O.I.) using the Revised Cochrane risk‐of‐bias tool for randomized trials (RoB 2) (Sterne et al. 2019) for RCTs, the Risk Of Bias In Non‐Randomized Studies—of Interventions (ROBINS‐I) (Sterne et al. 2016) for nonrandomized RCTs (nRCTs), and the Good Research for Comparative Effectiveness (GRACE) checklist (Dreyer et al. 2016) for observational studies.

### 2.5 Data Synthesis and Statistical Analysis

A descriptive summary of factors associated with chronic insomnia was presented.

Additionally, a series of assessments were conducted to determine the feasibility of conducting meta‐analysis and/or network meta‐analysis (NMA) to get a quantitative overview of long‐term BZD use in chronic insomnia. Of the studies included in the SLR, those reporting common outcomes were selected (e.g., studies assessing depressive symptoms via a common measure). The feasibility assessment consisted of a comparison across studies of their design (e.g., if both were RCTs), inclusion and exclusion criteria (looking for overall similarities or notable differences), criteria used to define insomnia, baseline characteristics, as well as availability and definitions of outcomes.

If studies reporting on the same outcomes had similar study and population characteristics based on a clinical review, a meta‐analysis and/or NMA was deemed relevant. Based on data availability and comparators reported across studies, frequentist pairwise meta‐analyses or NMAs were performed following Cochrane recommendations (Deeks et al. 2019). Both fixed‐ and random‐effect models were used, and the best fit was selected based on suspected heterogeneity, number of studies, and DIC criteria. Heterogeneity was assessed using the I^2^ statistic and Cochrane's Q test, and was suspected if either the test statistic was significant at 5% level or I^2^ > 50% (Higgins and Thompson 2002). For NMAs, loop inconsistency was assessed using Q‐statistics (Higgins et al. 2003). Inconsistency was suspected if the statistical test was significant at 5%. Meta‐analyses were conducted using R (v4.1.1) (R Core Team 2021) and obtained by the weighted average method using the R package *metafor* (Viechtbauer 2022). NMAs were also conducted using R through the *netmeta* package (Rücker et al., n.d.).

## 3 Results

### 3.1 Results of the Search

The total number of records retrieved using the search strategy and the studies identified as relevant at each step of the SLR screening for the two phases of the search are as shown in Figure 1 (for the detailed PRISMA flow diagram per phase of search, see Supporting Information Figure 1). After de‐duplication and screening of all retrieved studies, a total of 28 publications from 27 studies were included in the SLR. The most common reason for study exclusion upon full‐text review was outcomes not being of interest, with the next being intervention not of interest, primarily driven by BZD use of less than 3 months duration, and population not of interest.

**FIGURE 1:**
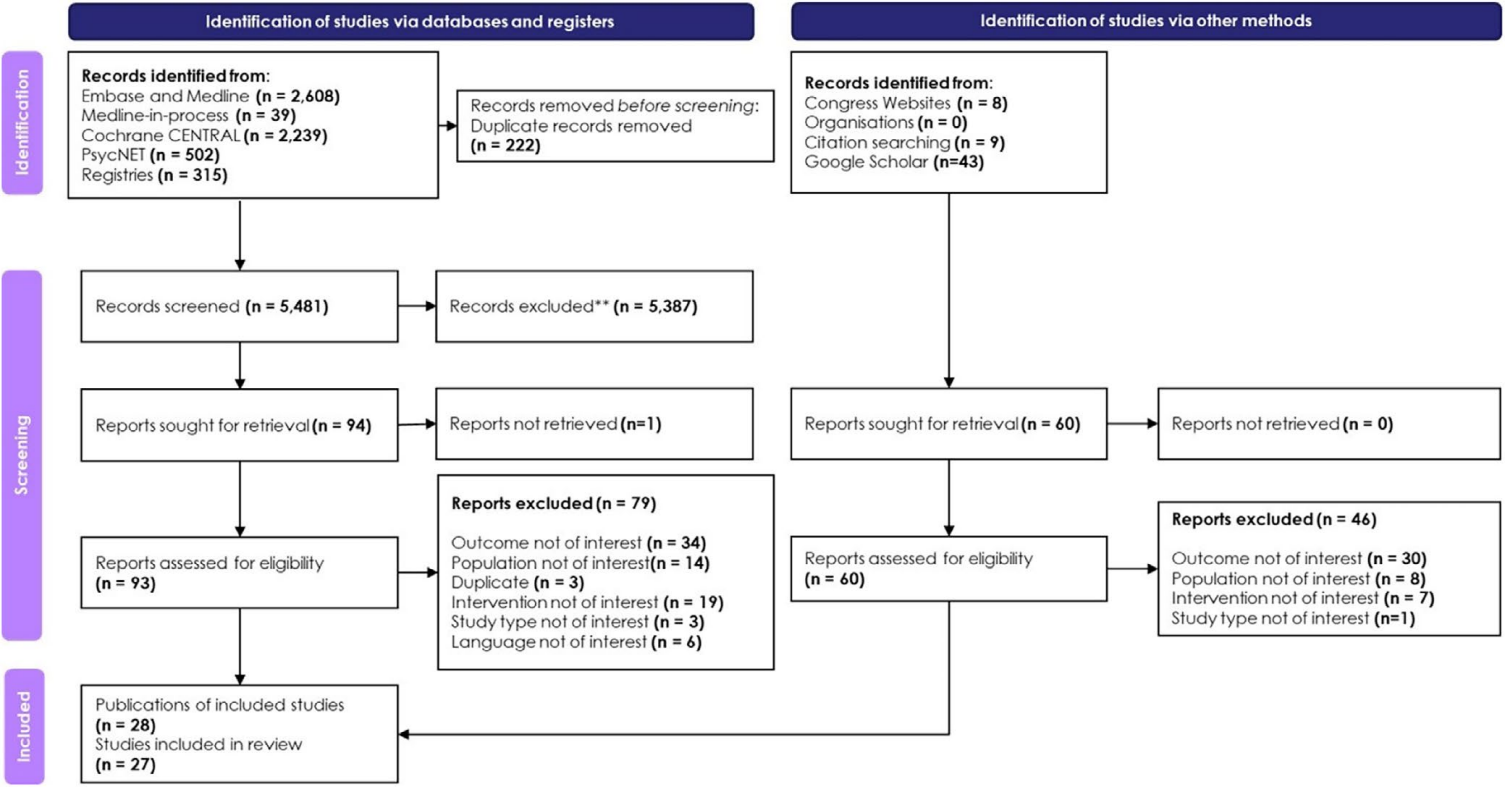
PRISMA flow diagram of the overall SLR process. PRISMA, Preferred Reporting Items for Systematic Reviews and Meta‐Analyses; SLR, systematic literature review.

### 3.2 Characteristics of Included Studies

The 28 publications from the 27 studies identified in the SLR were reviewed and considered for the qualitative overview and feasibility assessment of (network) meta‐analyses.

The publication by Bastien, Fortier‐Brochu, et al. (2003) is a sub study of Vignola et al. (2000) with the two publications reporting results from the same sample of study participants. Out of the 27 primary publications, studies were of prospective cohort (*n* = 8) (Bastien, Fortier‐Brochu, et al. 2003; Vignola et al. 2000; Jiang et al. 2019; Kudoh et al. 2004; Mazza et al. 2014; Schenck and Mahowald 1996; Schredl et al. 1998; Shash et al. 2016), retrospective cohort (*n* = 8) (Fang et al. 2009; Schonmann et al. 2018; Sato et al. 2020; Miano et al. 2020; Lavigne et al. 2019; Chung et al. 2015; Choi et al. 2018; Chen et al. 2012), cross‐sectional (*n* = 3) (Eto et al. 1998; Mah and Upshur 2002; Yen et al. 2015), RCT (*n* = 3) (Song et al. 2020; Baillargeon et al. 2003; Declerck and Smits 1999), nRCT (*n* = 3) (Poyares et al. 2004; Schneider‐Helmert 1988; Shapiro et al. 1995), and prospective case control (*n* = 2) (Guo et al. 2021; Bang et al. 2022) design. The most common geographical locations in the included studies were Canada (*n* = 5) (Bastien, Fortier‐Brochu, et al. 2003; Vignola et al. 2000; Mah and Upshur 2002; Baillargeon et al. 2003; Shapiro et al. 1995) and Taiwan (*n* = 4) (Fang et al. 2009; Chung et al. 2015; Chen et al. 2012; Yen et al. 2015). A summary of the included studies is shown in Supporting Information Table 9.

Twenty out of 28 included publications reported disaggregated baseline characteristics for patients with insomnia taking BZDs, as presented in Table 2. The largest population size per study was reported by Schonmann et al. (2018) as 154,929 patients, with the smallest population of only six patients reported in Mazza et al. (2014). The duration of insomnia in patients using BZD in the included studies ranged from 3.0 months in Song et al. (2020) to over 17.5 years in Bastien, LeBlanc, et al. (2003). The most common criterion used for chronic insomnia diagnosis in the included studies was from the DSM (III/IV/5). The mean age of patients with chronic insomnia taking BZDs ranged from 39.0 years in Song et al. (2020) to 77.9 years in Eto et al. (1998). The included studies reported a mixed distribution of gender with the male proportion ranging from 16.0% in Poyares et al. (2004) to 90.5% in Lavigne et al. (2019). Only one study from the USA reported racial distribution of patients with insomnia taking BZDs with around 77% of the study subjects being white (Lavigne et al. 2019). Two studies reported the household income of patients with insomnia taking BZDs, with around 60% of the study subjects earning a “low” income in one study (Guo et al. 2021) and 70% of the patients being retired in the other study (Bastien, Fortier‐Brochu, et al. 2003).

**TABLE 2:** Baseline characteristics of studies included in the SLR (27 studies, 28 publications).

Study	Study design	Study location	Sample size, *n*	Diagnostic criteria for chronic insomnia	Length of BZD use	Intervention comparator	Outcomes assessed
(Baillargeon et al. 2003)	RCT	Canada	65	≥ 6 months American Sleep Disorders Association	> 3 months	BZD tapering, BZD tapering + CBT	Safety
(Bang et al. 2022)	Prospective case–control study	South Korea	84	DSM‐5	> 180 days in a 1‐year period	Drug‐free insomnia, Healthy control	Safety, Sleep quality, Mental health
(Bastien, LeBlanc, et al. 2003)	Prospective	Canada	46	≥ 6 months ICSD and DSM‐IV	NR	Drug‐free insomnia patients, Good sleepers	Mental health, Sleep quality
(Chen et al. 2012)	Retrospective	Taiwan	34,158	ICD‐9‐CM	Long‐term use, Prescribed ≥ 30 DDD/year	Non‐BZD + BZD, Non‐BZD	Safety
(Choi et al. 2018)	Retrospective	South Korea	500,959	ICD‐10‐CM	≥ 30 DDD of sedative‐hypnotics per year and followed for 11 years	BZD + zolpidem, Zolpidem, No exposure	Safety
(Chung et al. 2015)	Retrospective	Taiwan	30,670	ICD‐9‐CM	Long‐term use	Zolpidem, Zopiclone, Zaleplon	Mental health
(Declerck and Smits 1999)	RCT	Netherlands	22	Subjective inability to obtain or maintain adequate sleep is termed chronic insomnia	NR	Insomnia patients switched to zolpidem	Mental health, Sleep quality
(Eto et al. 1998)	Cross‐sectional	Japan	53	NR	NR	Ambulant elderly patients not taking any hypnotics or psychotropics, BZD at night	Safety
(Fang et al. 2019)	Retrospective	Taiwan	43,585	ICD‐9‐CM	≥ 30 DDD of sedative‐hypnotics per year with < 3 months between prescriptions	Zolpidem, Zopiclone, Zaleplon	Safety
(Guo et al. 2021)	Prospective case–control study	China	120	DSM‐5	NR	Zolpidem, Zopiclone	Safety, Mental health, Sleep quality
(Jiang et al. 2019)	Prospective	China	605	Athens Insomnia Scale score ≥ 6, self‐reported information	≥ 1 year	Zolpidem, Eszopiclone, Zaleplon, Traditional Chinese medicines	Safety
(Kudoh et al. 2004)	Prospective	Japan	328	Insomnia not defined	Long‐term daily BZD use for > 1 year	BZDs for anxiety and/or depression	Safety, Mental health
(Lavigne et al. 2019)	Retrospective	USA	348,449	NR	Received treatment for insomnia for 2 years	Zolpidem, Trazodone, Antihistamine	Safety, Mental health
(Mah and Upshur 2002)	Cross‐sectional	Canada	118	NR	≥ 1 year	Physicians comprising sleep specialists, family physicians, and family medicine residents	QoL
(Mazza et al. 2014)	Prospective	Italy	61	Based on BZD use which were prescribed for chronic insomnia	NR	Healthy subjects	Mental health, Sleep quality
(Miano et al. 2020)^a^	Retrospective	NR	34	Unspecified, primary chronic insomnia	Long‐term use of high doses of BZDs	Control	Safety
(Poyares et al. 2004)	nRCT	Brazil	43	DSM‐IV	NR	Healthy controls	Safety, Sleep quality
(Sato et al. 2020)	Retrospective	Japan	2213	Unspecified clinical diagnosis based on ICSD‐2	NR	Zopiclone, Zolpidem, Eszopiclone	Safety
(Schenck and Mahowald 1996)	Prospective	USA	170	DSM‐III or DSM‐III‐R	≥ 6 months	Patients with other sleep disorders	Safety, Mental health
(Schneider‐Helmert 1988)	nRCT (open‐label)	Switzerland	76	Persistent psychophysiological insomnia not primarily due to physical or psychiatric illness, drugs or alcohol	≥ 6 months	Drug‐free insomnia patients	Safety, Sleep quality
(Schonmann et al. 2018)	Retrospective	Israel	236,597	NR	Long‐term use, ≥ 1 year	Zopiclone, Zolpidem	Safety
(Schredl et al. 1998)	Prospective	Germany	152	Primary insomnia, common diagnostic criteria^b^	BZD for primary insomnia	Antidepressants and drug‐free insomnia patients	Safety
(Shapiro et al. 1995)	nRCT	Canada	134	NR	NR	Insomnia patients switched to zopiclone	Safety
(Shash et al. 2016)	Prospective	France	9294	NR	Association of BZD use and dementia over 8 years	BZD‐free insomnia patients	Safety
(Song et al. 2020)	RCT	China	207	DSM‐5	≥ 3 months	Lorazepam + JW‐SZRD	Mental health, QoL, Sleep quality
Primary study: (Vignola et al. 2000) Secondary study: (Bastien, Fortier‐Brochu, et al. 2003)	Prospective cohort	Canada	60	DSM‐IV	NR	Drug‐free insomnia patients, Good sleepers	Safety, Mental health, Sleep quality
(Yen et al. 2015)	Cross‐sectional	Taiwan	139	Received treatment for insomnia	NR	Zolpidem	Safety

Abbreviations: BZD, benzodiazepine; CBT, cognitive behavioural therapy; DDD, defined daily dose; DSM, Diagnostic and Statistical Manual of Mental Disorders; ICD‐10‐CM, International Classification of Diseases, Tenth Revision, Clinical Modification; ICD‐9‐CM, International Classification of Diseases, Ninth Revision, Clinical Modification; JW‐SZRD, Jiawei Suanzaoren Decoction; *n*, number; NR, not reported; nRCT, nonrandomized controlled trial; QoL, quality of life; RCT, randomized controlled trial; SD, standard deviation.

^a^ This study was an abstract.

^b^ Common diagnostic criteria included: Deutsches Institut für Medizinische Dokumentation und Information 1994; Schramm and Riemann 1995; and American Psychiatric Association 1994.

### 3.3 BZDs And Their Comparators in the Included Studies

All included studies reported the duration of BZD use as at least 3 months, with only 11 studies reporting a mean duration ranging from 3.1 to 13.5 years. Twenty‐five types of BZDs were reported in the included studies: alprazolam, bromazepam, brotizolam, chlorazepam, chlordiazepam, chlordiazepoxide, clobazam, clonazepam, clorazepate, clotiazepam, diazepam, estazolam, ethyl loflazepate, fludiazepam, flunitrazepam, flurazepam, lorazepam, lormetazepam, medazepam, midazolam, nitrazepam, nordazepam, oxazepam, temazepam, and triazolam. All studies had a comparator to the BZD arm, with many of the included studies reporting more than one comparator. The most examined comparators among the included publications were Z‐drugs (*n* = 9 publications), drug‐free patients with insomnia (*n* = 7), healthy controls or good sleepers (*n* = 6), a combination of Z‐drugs and BZDs (*n* = 2), and patients with insomnia who switched to Z‐drugs from BZDs (*n* = 2).

### 3.4 Risk of Bias in Included Studies

From the three RCTs, one RCT was associated with a high risk of bias and the other two with, respectively, a low risk of bias and some concerns for bias. The RCTs were primarily degraded for concerns regarding randomization, allocation concealment, and blinding. The three nRCTs were all assessed as at serious risk of bias due to the inability of study authors to adjust results for potential confounders. From the 21 observational studies, 19 were considered as of good quality while the remaining two were assessed as of fair quality. The observational studies primarily reported a lack of adjustment of confounding variables and unclear information as to whether the comparators in the study were initiated simultaneously.

### 3.5 Study Outcomes

Most of the publications reported safety outcomes related to BZD use in chronic insomnia (*n* = 22) (Bastien, Fortier‐Brochu, et al. 2003; Vignola et al. 2000; Jiang et al. 2019; Kudoh et al. 2004; Schenck and Mahowald 1996; Schredl et al. 1998; Shash et al. 2016; Schonmann et al. 2018; Sato et al. 2020; Miano et al. 2020; Lavigne et al. 2019; Choi et al. 2018; Chen et al. 2012; Eto et al. 1998; Yen et al. 2015; Baillargeon et al. 2003; Poyares et al. 2004; Schneider‐Helmert 1988; Shapiro et al. 1995; Guo et al. 2021; Bang et al. 2022; Fang et al. 2019). This was followed by mental health outcomes (*n* = 11) (Bastien, Fortier‐Brochu, et al. 2003; Vignola et al. 2000; Kudoh et al. 2004; Mazza et al. 2014; Schenck and Mahowald 1996; Lavigne et al. 2019; Chung et al. 2015; Song et al. 2020; Declerck and Smits 1999; Guo et al. 2021; Bang et al. 2022), sleep quality (*n* = 9) (Bastien, Fortier‐Brochu, et al. 2003; Vignola et al. 2000; Mazza et al. 2014; Song et al. 2020; Declerck and Smits 1999; Poyares et al. 2004; Schneider‐Helmert 1988; Guo et al. 2021; Bang et al. 2022), and QoL (*n* = 2) (Mah and Upshur 2002; Song et al. 2020). Overall, the included studies demonstrated inconsistent evidence of outcomes associated with long‐term use of BZDs, particularly with types of outcomes measured and follow‐up durations for the reported outcomes. Furthermore, only 20 of 28 included publications reported disaggregated baseline characteristics for patients with insomnia taking BZDs. Due to these limitations, the feasibility assessment for meta‐analyses or NMAs were able to identify only five suitable outcomes classified within the aforementioned outcomes categories, as follows: insomnia severity and overall sleep quality (sleep quality outcomes), incidence of falls (safety outcome), and depression and anxiety symptom severity (mental health outcomes).

#### 3.5.1 Sleep Quality Outcomes

Nine studies (10 publications) (Bastien, Fortier‐Brochu, et al. 2003; Vignola et al. 2000; Mazza et al. 2014; Song et al. 2020; Declerck and Smits 1999; Poyares et al. 2004; Guo et al. 2021; Bang et al. 2022; Bastien, LeBlanc, et al. 2003; Alqurashi et al. 2021) reported sleep quality outcomes in patients with chronic insomnia taking BZDs over the long term, where selected studies assessed sleep quality based on self‐report measures, including the Insomnia Severity Index (ISI) (Bastien, Fortier‐Brochu, et al. 2003; Song et al. 2020; Guo et al. 2021; Bang et al. 2022), Sleep Impairment Index (SII) (Vignola et al. 2000), Pittsburgh Sleep Quality Index (PSQI) (Mazza et al. 2014; Guo et al. 2021), Stanford sleepiness scale (SSS) (Song et al. 2020), Groningen sleep scales (GSS) (Declerck and Smits 1999), Epworth Sleepiness Scale (ESS) (Mazza et al. 2014; Alqurashi et al. 2021), and Visual analogue scale (VAS) (Alqurashi et al. 2021). Of these, the ISI and PSQI scales provided the most relevant assessment of insomnia and overall sleep quality compared to the SSS, GSS, and ESS, which focus primarily on daytime functioning and sleepiness. As well, the ISI and PSQI have been the recommended measures for insomnia symptoms and global sleep quality, respectively, in a standard research assessment protocol for insomnia and were reported in several studies included in this SLR (Buysse et al. 2006). Although the VAS also provides a comprehensive assessment of sleep‐related concerns, including alertness, energy, and mood, only the Vignola et al. (2000) study reported on this assessment. As such, the current SLR presents findings, in subsequent sections, from the studies reporting ISI and PSQI scores in additional details to assess feasibility of performing meta‐analyses to support the overall review.

Physiological measures of sleep, such as sleep latency time, total sleep time, and WASO were additionally assessed by six studies (seven publications), although there was considerable variation in the populations being compared (e.g., comparisons between use of BZD and other medications, between use of BZD and no medication, between patients with insomnia and good sleepers) (Vignola et al. 2000; Mazza et al. 2014; Song et al. 2020; Declerck and Smits 1999; Poyares et al. 2004; Schneider‐Helmert 1988; Bastien, LeBlanc, et al. 2003). These outcomes, as well as SSI, SSS, GSS, ESS and VAS have been reported in detail in Supporting Information Table 10. Overall, when comparing patients with insomnia using BZDs to good sleepers, one study found that long‐term BZD use was associated with shorter (i.e., better) sleep latency time but similar sleep quality on other measures, while two others found overall poorer sleep quality across all measures, when compared to good sleepers (Vignola et al. 2000; Mazza et al. 2014; Poyares et al. 2004).

##### 3.5.1.1 ISI

Findings from the ISI, assessing severity of insomnia symptoms, were reported by three studies: Bastien, Fortier‐Brochu, et al. (2003), Song et al. (2020), and Bang et al. (2022). A feasibility assessment was conducted for a meta‐analysis on ISI scores based on these three studies, with relevant study details displayed in Table 3.

**TABLE 3:** Overview and baseline characteristics of studies included in feasibility assessment for potential meta‐analyses and NMAs (10 studies).

Study	Insomnia definition	Key inclusion/exclusion criteria	Treatment arm	Sample size, *n*	Duration of chronic insomnia: Mean (IQR)	Age, mean (SD)	Males, %	Time of assessment	Treatment duration, mean (SD) in years
(Bang et al. 2022)	DSM‐5	*Inclusion*: Age ≥ 55 years *Exclusion*: NR	Chronic insomnia, BZD use	29	NR	68.03 (8.38)	34.50%	NR	Median: 11.8 years
			Chronic insomnia, drug‐free	27	NR	64.89 (6.94)	14.80%	NR	NA
			Age‐ and sex‐matched controls	28	NR	64.56 (6.48)	28.60%	NR	NA
(Bastien, LeBlanc, et al. 2003)	DSM‐IV	*Inclusion*: Age ≥ 55 years Insomnia for > 3 nights/week for > 6 months ≥ 1 daytime consequence due to insomnia *Exclusion*: NR	Chronic insomnia, BZD use	15	17.5 years (SD = 8.3)	62.2 (6)	46.70%	0 days (on BZD for > 3 months)	13.5 (10)
			Chronic insomnia, drug‐free	15	27.8 years (SD = 17.7)	63.4 (5.4)	53.40%	0 days (on BZD for > 3 months)	NA
			Good sleepers	16	NA	63.1 (6.32)	56.20%	0 days (on BZD for > 3 months)	NA
(Eto et al. 1998)	Taking benzodiazepines as hypnotics mostly every night for more than 1 month	*Inclusion*: Elderly patients, Living in a long‐term care facility *Exclusion*: NR	BZD users at night	25	NR	77.9 (5.8)	32%	NR	3.71 (0.11–14.75)
			Rehab patients not taking any hypnotics or psychotropic drugs	28	NR	78.4 (6.3)	35%	NR	NA
(Guo et al. 2021)	DSM‐5	*Inclusion*: Age 50–80 years, Insomnia for > 3 nights/week for > 6 months, ≥ 1 daytime consequence due to insomnia *Exclusion*: Significant medical, neurological disorder, Excl. major psychopathology	BZD use	30	17 years (5–34)	64.2 (6.98)	20.83%	NR	8.92 (12.39)
			Z‐drug use	90	10 years (3–15.25)	59.22 (5.52)	20.83%	NR	
(Jiang et al. 2019)	Athens Insomnia Scale score ≥ 6 indicated insomnia within the previous month, then patients were followed up for 1 year (8‐item scale with a total score ranging between 0 and 24)	*Inclusion*: Elderly patients *Exclusion*: NR	Insomniacs using BZD	19	NR	84.33 (NR)	12.85%	Within the 1st year of follow‐up	NA
			Insomniacs, not using hypnotics	83	NR	77.9 (5.8)	32%	Within the 1st year of follow‐up	NA
			Insomniacs, not using BZD	18	NR	78.4 (6.3)	35%	Within the 1st year of follow‐up	NA
(Lavigne et al. 2019)	Patients who received a prescription of medication for insomnia	*Inclusion*: Age > 18 years, ≥ 1 medication other than insomnia *Exclusion*: Suicide attempt in 12 months prior to first insomnia medication prescription	BZD matched with zolpidem	72,641	NR	59.5 (15.5)	90.50%	NR	3017 (5.2%)
			BZD	83,795	NR	61.7 (15.6)	91.10%		3759 (4.5%)
(Mazza et al. 2014)	DSM‐IV	*Inclusion*: NR *Exclusion*: Major psychopathology	BZD abuse	6	NR	53.3 (14.8)	66.7%	NR	3.5 (range: 2–6)
(Schenck and Mahowald 1996)	DSM‐III‐R	*Inclusion*: Adults, Referrals for ≥ 6 months with nightly benzodiazepine therapy for longstanding, sleep‐disruptive disorders *Exclusion*: NR	Long‐term users BZD	25	17.5 years (SD = 8.3)	NR	36%	NR	1 (4%) (in remission)
(Song et al. 2020)	DSM‐5	*Inclusion*: Age 18–60 years, Patients with primary insomnia, chronic insomnia, and insomnia disorder *Exclusion*: Secondary insomnia caused by other disease, Abnormal liver function	Control (lorazepam)	94	NR	39.3 (9.63)	33%	Weeks 0, 4, 8, 12	NR
			Treatment (JW‐SZRD + lorazepam)	113	NR	44 (10.45)	34.5%	Weeks 0, 4, 8, 12	NR
(Vignola et al. 2000)	DSM‐IV and ICSD‐10	*Inclusion*: Age > 55 years *Exclusion*: Significant medical, neurological disorder, Excl. major psychopathology	Drug‐free individuals with insomnia	20	25.8 years (SD = 15.7)	61.7 (6.43)	50%	2nd and 3rd night	NA
			Individuals with insomnia using BZD	20		62.3 (5.89)	45%	2nd and 3rd night	13.5 (10)
			Self determined good‐sleepers	20	NA	63.35 (5.15)	55%	2nd and 3rd night	NA

Abbreviations: BZD, benzodiazepine; DSM, Diagnostic and Statistical Manual of Mental Disorders; ICD‐10‐CM, International Classification of Diseases, Tenth Revision, Clinical Modification; IQR, interquartile range; JW‐SZRD, Jiawei Suanzaoren Decoction; *n*, number; NA, not applicable; NMA, network meta‐analysis; NR, not reported; SD, standard deviation.

All three publications included study arms of patients with chronic insomnia taking and not taking a BZD (with Song et al. (2020) specifying treatment with Jiawei Suanzaoren Decoction (JW‐SZRD) and lorazepam), as well as a healthy control arm (Bastien, Fortier‐Brochu, et al. 2003; Song et al. 2020; Bang et al. 2022). When comparing inclusion and exclusion criteria reported by each study, Bastien, LeBlanc, et al. (2003) and Song et al. (2020) included criteria to recruit patients with genuine insomnia and not related to substance use or psychological disorder. There was also a short overlap in age criteria, where Song et al. (2020) reported a lower age cutoff compared to the other two studies (Bastien, Fortier‐Brochu, et al. 2003; Bang et al. 2022).

Looking at baseline characteristics, there were key differences between studies, particularly in mean age and proportion of males included in the studies. Further differences were seen in treatment duration, where patients reported by Bang et al. (2022) and Bastien, LeBlanc, et al. (2003) were treated on average for more than 10 years, whereas subjects from Song et al. (2020) were treated for 12 weeks. Based on these differences that may create bias on estimates and interpretation, Song et al. (2020) was excluded from the NMA.

Findings from the NMA performed with data from Bang et al. (2022) and Bastien, LeBlanc, et al. (2003) (Figure 2A) showed that the use of BZDs in patients with insomnia was significantly associated with a lower ISI score (i.e., lower insomnia severity) compared to drug‐free patients (mean difference (MD): −3.67 (95% confidence interval (CI) −5.97 to −1.37), Figure 2B) and significantly higher insomnia severity compared to good sleepers (11.95 (95% CI 10.04 to 13.87), Figure 2B). These findings suggest that long‐term use of BZDs helps reduce insomnia symptoms among patients compared to not being treated but does not improve the severity of insomnia symptoms to a degree comparable to that of individuals without chronic insomnia.

**FIGURE 2:**
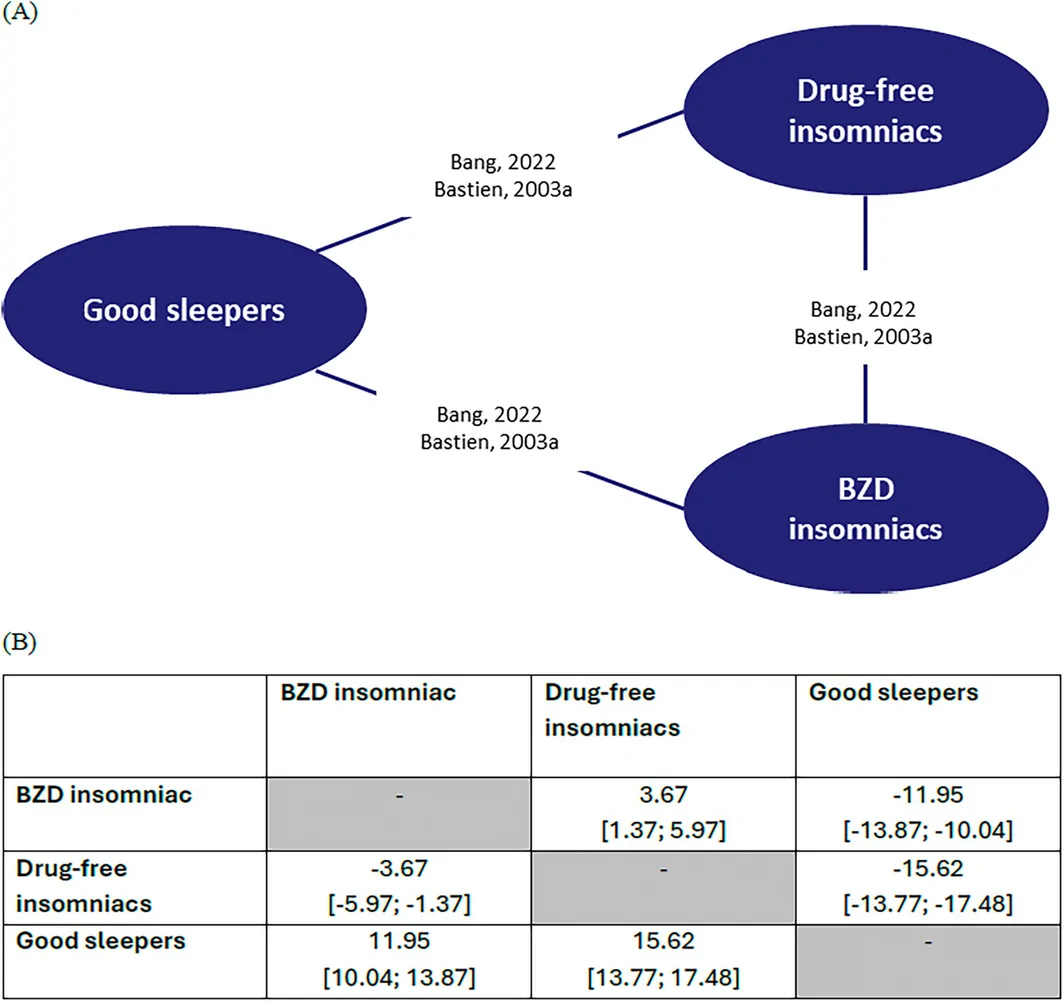
Network (A) and league table (B) of the NMA conducted on ISI. ISI, Insomnia Severity Index; NMA, network meta‐analysis.

##### 3.5.1.2 PSQI

Scores from the PSQI were reported by only two studies: Mazza et al. (2014) and Guo et al. (2021), with relevant study details presented in Table 3. Commonalities between the two studies were in design (both prospective observational studies) and study population, which consisted of patients with chronic insomnia (Mazza et al. 2014; Guo et al. 2021). However, Mazza et al. (2014) had only a BZD use arm, whereas Guo et al. (2021) also included a non‐BZD use arm.

There was a limited overlap in the inclusion and exclusion criteria for the two studies. Both studies excluded patients with a major psychopathology; however, Mazza et al. (2014) did not expand further on criteria (Mazza et al. 2014; Guo et al. 2021).

Examination of baseline characteristics also demonstrated some differences, specifically in sample size and proportion of males included, but patients were similar in age (Mazza et al. 2014; Guo et al. 2021). Further differences were seen in study results where treatment duration was 3.5 years in the Mazza et al. (2014) study and 8.92 years in the Guo et al. (2021) study. Due to the overlap of only one study arm, a meta‐analysis of patients with insomnia treated with BZDs was performed to obtain a pooled mean PSQI score.

Given the findings from the feasibility assessment, the meta‐analysis was conducted but results are limited to interpretation due to heterogeneity. Results showed a pooled mean PSQI score of 13.81 (95% CI 12.78 to 14.85; Figure 3) with high statistical heterogeneity (*p* = 0.01, I^2^ = 85%). Although this is comparable to the observed mean PSQI score of patients taking no medications in the Guo et al. (2021) study (i.e., 13.90) and could suggest that patients with chronic insomnia have a similar sleep quality regardless of BZD use.

**FIGURE 3:**
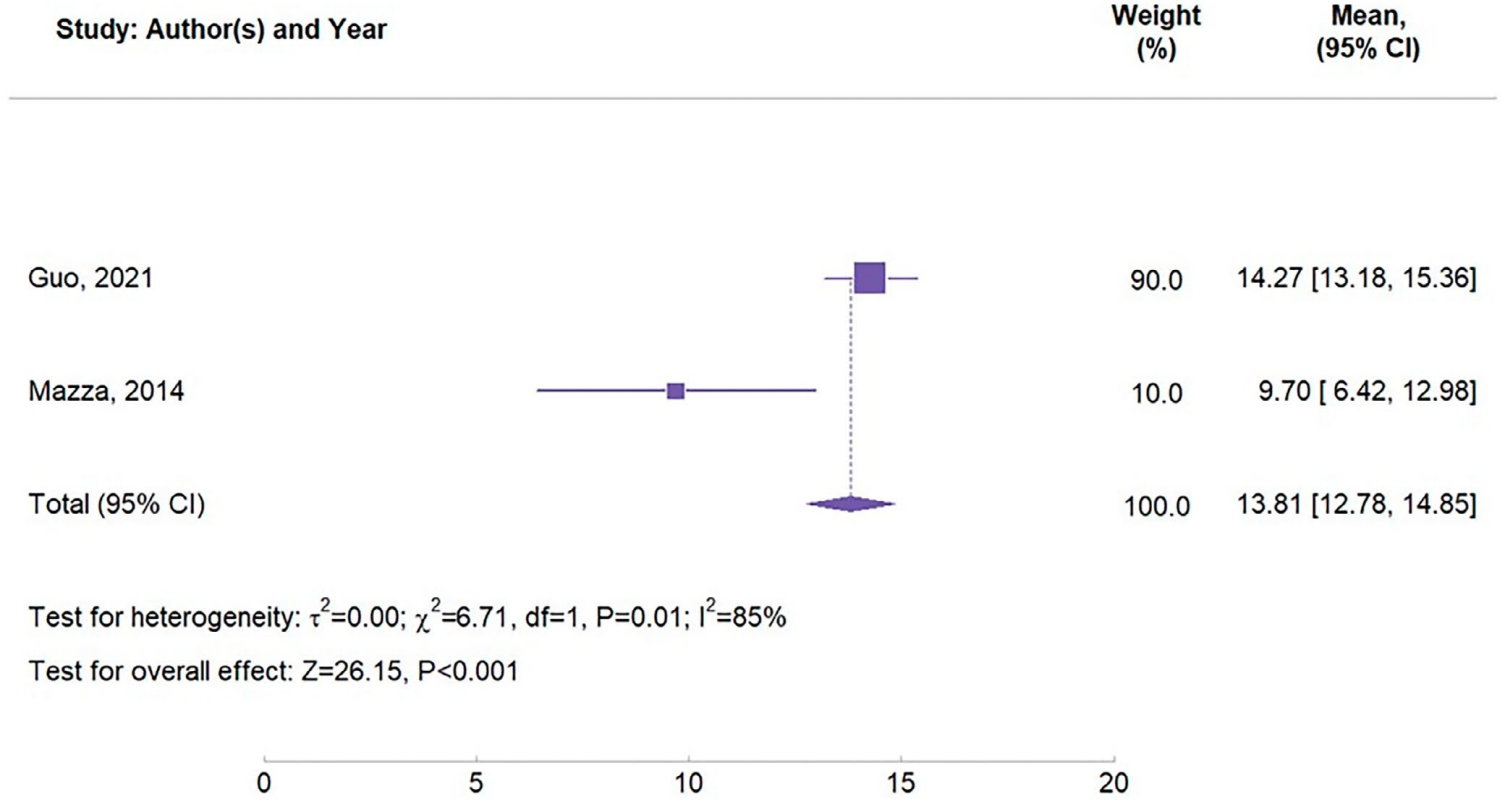
Forest plot of meta‐analysis on patients with insomnia using BZD subjects for PSQI. BZD, benzodiazepine; PSQI, Pittsburgh Sleep Quality Index.

#### 3.5.2 Safety Outcomes

Among the safety outcomes reported in 21 studies (22 publications) included in this SLR, only a few were reported similarly in more than one study: BZD dependence, abuse, or addiction, cognitive impairment, dementia risk, falls, and mortality, described in further detail below (Bastien, Fortier‐Brochu, et al. 2003; Vignola et al. 2000; Jiang et al. 2019; Kudoh et al. 2004; Schenck and Mahowald 1996; Schredl et al. 1998; Shash et al. 2016; Schonmann et al. 2018; Sato et al. 2020; Miano et al. 2020; Lavigne et al. 2019; Choi et al. 2018; Chen et al. 2012; Eto et al. 1998; Yen et al. 2015; Baillargeon et al. 2003; Poyares et al. 2004; Schneider‐Helmert 1988; Shapiro et al. 1995; Guo et al. 2021; Bang et al. 2022; Fang et al. 2019). Other outcomes, such as gait and balance profile, cancer risk, and withdrawal symptoms, were reported in one study each and/or using different outcome measures and were, therefore, not able to be synthesized (Kudoh et al. 2004; Eto et al. 1998; Poyares et al. 2004; Schneider‐Helmert 1988; Shapiro et al. 1995; Fang et al. 2019). As such, findings related to these outcomes, along with the ones described below, are described in Supporting Information Table 11.

##### 3.5.2.1 Dependence, Abuse, or Addiction

Dependence, abuse, or addiction (of alcohol or substances) were respectively reported in five studies (Schenck and Mahowald 1996; Schonmann et al. 2018; Lavigne et al. 2019; Yen et al. 2015; Schneider‐Helmert 1988). However, definitions used across studies for these outcomes differed substantially, and none provided comparison between individuals taking BZD and good sleepers, preventing from drawing general conclusions.

Regarding alcohol abuse, both Lavigne et al. (2019) and Schenck and Mahowald (1996), reported similar rates among patients taking a BZD (4.5% and 4%, respectively). As such, a feasibility assessment of these studies was conducted but found that notable differences were present in study design and baseline characteristics of study participants (see Table 3 for more details). Lavigne et al. (2019), had four cohorts, consisting of patients with chronic insomnia treated with a BZD, with zolpidem, with trazodone, or with antihistamine. Schenck and Mahowald (1996), however, had two subgroups: patients who were and were not taking a BZD. As well, Lavigne et al. (2019), defined chronic insomnia as having a prescription for a medication for insomnia, whereas Schenck and Mahowald (1996), used the DSM‐III‐R description to define chronic insomnia. There was no overlap of the minimal inclusion/exclusion criteria reported, aside from age group, which demonstrated both studies included adult participants, but with Lavigne et al. 2019, including veterans only (Schenck and Mahowald 1996; Lavigne et al. 2019).

Study participants were almost exclusively male in the Lavigne et al. (2019) study (90.5% to 91.1%) but males represented only 36% of the Schenck and Mahowald (1996) population. Added to this, sample sizes differed substantially between studies, with the Lavigne et al. (2019) study having 83,795 participants treated with a BZD and the Schenck and Mahowald (1996) study having only 25 individuals taking BDZ. Time of assessment was not reported by either study, and treatment duration was not provided by Schenck and Mahowald (1996) and Lavigne et al. (2019).

Taken together, it was determined that a meta‐analysis could be conducted but was not recommended due to the difference in sample sizes between the studies, which would result in the Lavigne et al. (2019) study, with the larger sample size, being overrepresented in the results compared to the Schenck and Mahowald (1996) study.

##### 3.5.2.2 Cognitive Impairment and Dementia

Eight studies (nine publications) reported cognitive outcomes, including two which reported on dementia risk (Bastien, Fortier‐Brochu, et al. 2003; Vignola et al. 2000; Kudoh et al. 2004; Schredl et al. 1998; Shash et al. 2016; Miano et al. 2020; Chen et al. 2012; Guo et al. 2021; Bang et al. 2022). A study by Miano et al. 2020, showed significant impairment of global cognition and several cognitive domains, including memory, executive function, and processing speed, in patients with chronic insomnia taking high doses of BZD over the long term. Likewise, Bang et al. 2022, found that patients with chronic insomnia using BZDs on the long‐term showed a significant decrease in score on the Trail Making Test—Part A (TMT‐A), measuring processing speed, compared to drug‐free insomnia patients and controls (0.73 ± 0.66 vs. 1.27 ± 0.38 vs. 1.09 ± 0.47), and in categorical fluency, measuring language and executive functioning than drug‐free insomnia patients (−0.01 ± 0.99 vs. 1.26 ± 0.97).

Both Guo et al. (2021) and Vignola et al. (2000) reported the Mini Mental State Examination (MMSE) score and Trail Making Test—Part B (TMT‐B). While studies were similar in terms of study type (i.e., prospective observational design), inclusion of patients of similar age, and exclusion of patients with significant medical, neurological, and major psychopathological disorders, several differences were identified, including the definitions used for insomnia (DSM‐5 in Guo et al. (2021) versus DSM‐IV and ICSD‐10 in Vignola et al. 2000) and treatment duration. These differences as well as availability of median values as opposed to mean scores led to no meta‐analysis conducted on these outcomes given associated limitations that could lead to biased estimates.

Reporting results from the same control population, Bastien, LeBlanc, et al. (2003) and Vignola et al. (2000), reported a correlational analysis which revealed that physiological and subjective measures of daytime performance of memory and global cognition are significantly related to sleep quality for the three groups. The study showed that a good night's sleep assessed physiologically can lead to better cognitive performance in good sleepers and insomnia patients (Bastien, Fortier‐Brochu, et al. 2003; Vignola et al. 2000). Likewise, Kudoh et al. (2004) described the incidence of postoperative confusion to be significantly higher in long‐term (daily use for more than 1 year) than in short‐term BZD users or non‐users. However, Schredl et al. (1998) showed BZD use to have no effect on dream recall frequency. Similarly, withdrawal side effects were described by Schneider‐Helmert 1988, which showed reduced mental but not physical capacity after BZD withdrawal.

Patients with chronic insomnia using BZDs in Shash et al. (2016) reportedly had a 10% increased risk of dementia (hazard ratio (HR) 1.16 (95% CI 0.77 to 1.75)) compared to non‐users which can include drug‐free insomnia patients or Z‐drug insomnia patients. Patients taking BZDs with a longer half‐life (> 20 h) showed a further increased risk of dementia compared with short half‐life BZD users (Shash et al. 2016). Dementia risk was higher in patients with a history of hypnotic usage compared to those without (2.34 (95% CI 1.92 to 2.85), *p* < 0.001), as reported by Chen et al. (2012).

##### 3.5.2.3 Falls

Another safety outcome of interest was fall incidence, reported by Jiang et al. (2019) and Eto et al. (1998). These two studies were considered for a feasibility assessment for meta‐analysis. Both studies assessed subgroups with patients taking BZDs and patients not taking hypnotic medication. However, the studies differed in the definitions of insomnia: Jiang et al. (2019) used the Athens Insomnia Scale. Conversely, Eto et al. (1998) defined insomnia as taking BZDs as hypnotics most nights for at least 1 month. Overall, very few inclusion and exclusion criteria were reported and both studies exclusively included elderly patients (Jiang et al. 2019; Eto et al. 1998). However, differences in criteria were that Jiang et al. (2019) reported inclusionary criteria dependent on ambulation status and whether or not participants were taking hypnotic medications whereas Eto et al. (1998) specifically identified participants living in long‐term care facilities. Baseline characteristics were also limited and, when available, highlighted differences, particularly in sample size (*n* = 120 in Jiang et al. 2019 and *n* = 25–28 in Eto et al. 1998) and proportion of males included in the studies (12.85% in Jiang et al. 2019 and 32% in Eto et al. 1998). However, mean age across study arms of the two studies was similar, ranging from 77.9 to 78.4 years in Eto et al. (1998) to 84.33 years in Jiang et al. (2019). Overall, a NMA was deemed to be feasible to compare patients with chronic insomnia taking a BZD versus patients not taking BZD versus patients not taking a hypnotic medication, as reported by Jiang et al. (2019).

Both studies reported higher rates of falls for patients with insomnia using a BZD (36.7% and 32%, respectively) compared to patients not using hypnotic drugs (30.1% and 25%, respectively) and patients treated by non‐BZD drugs (33.3% in Jiang et al. 2019) (Jiang et al. 2019; Eto et al. 1998). Jiang et al. (2019) found a significant increased risk of falls in patients with insomnia, as well as in patients using BZDs after adjusting for potential confounders, while Eto et al. (1998) observed no differences in risk of falls in BZD users compared to non‐users. This result is consistent with findings from the NMA led on both studies (Figure 4A). Patients with insomnia using long‐term BZDs showed a higher but non‐significant risk of falls compared to patients not using hypnotic drugs and patients treated by non‐BZD drugs (OR 1.39 (95% CI 0.63 to 3.03) and 1.18 (0.34 to 4.08), respectively; see Figure 4B).

**FIGURE 4:**
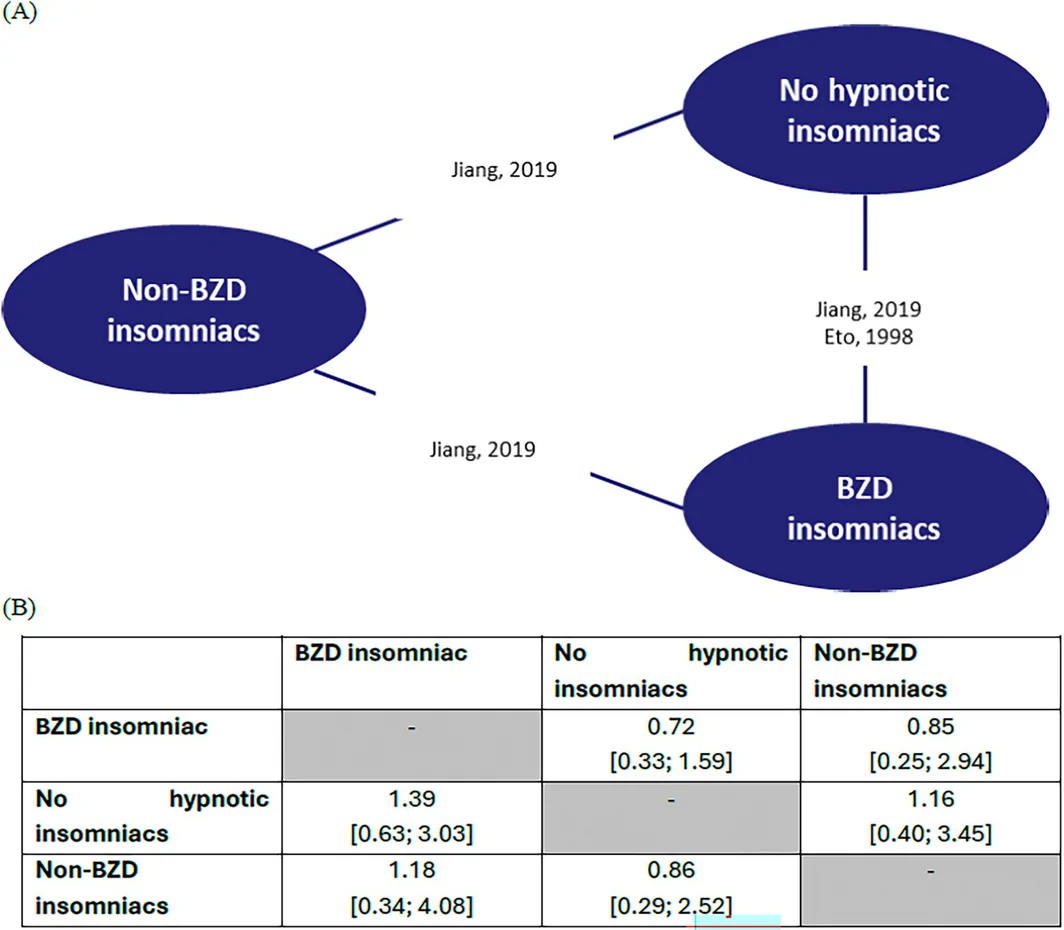
Network (A) and league table (B) of the NMA conducted on incidence of falls. NMA, network meta‐analysis.

##### 3.5.2.4 Mortality

Three studies reported mortality outcomes (Schonmann et al. 2018; Sato et al. 2020; Choi et al. 2018). However, no meta‐analysis was deemed feasible due to heterogeneity in terms of length of follow‐up and outcome definition.

#### 3.5.3 Mental Health Outcomes

Of the four studies (five publications) reporting on mental health outcomes, depression and anxiety, as reported by the Beck Depression Inventory (BDI) and Beck Anxiety Inventory (BAI), respectively, were the most widely reported outcomes, described below (Bastien, Fortier‐Brochu, et al. 2003; Vignola et al. 2000; Mazza et al. 2014; Bang et al. 2022; Bastien, LeBlanc, et al. 2003). Other outcomes which were reported in single studies each include suicide risk, rates of psychiatric disorders, and symptoms of depression or anxiety reported via other measures (i.e., Hamilton Depression Rating Scale and Hamilton Anxiety Rating Scale) (Schenck and Mahowald 1996; Lavigne et al. 2019; Chung et al. 2015; Song et al. 2020; Declerck and Smits 1999; Guo et al. 2021). Details from these six studies have been provided in Supporting Information Table 12.

##### 3.5.3.1 Depression

Bang et al. (2022), Bastien, LeBlanc, et al. (2003), Vignola et al. (2000), and Mazza et al. (2014) reported depressive symptoms assessed by the BDI. The four studies were entered into a feasibility assessment for a meta‐analysis (see Table 3 for relevant study details).

These prospective observational studies were conducted in South Korea (*n* = 1), Canada (*n* = 2), and Italy (*n* = 1) and generally included patients over 55 years of age with chronic insomnia (Bastien, Fortier‐Brochu, et al. 2003; Vignola et al. 2000; Mazza et al. 2014; Bang et al. 2022). The only group analysed in common across studies is patients with insomnia taking BZDs. Other subgroups assessed were patients with chronic insomnia not taking BZDs and healthy controls. Inclusion and exclusion criteria were reported in detail by Bastien, LeBlanc, et al. (2003) and Vignola et al. (2000) and were similar; however, Bang et al. (2022) and Mazza et al. (2014) reported very limited criteria. Baseline study characteristics were similar across all four studies, with notable differences being Bang et al. (2022) having a slightly lower proportion of males (14.8% to 34.5%) compared to the other three studies (45% to 66.7%) and Mazza et al. (2014) having a lower sample size (*n* = 6) compared to the other three studies (*n* = 15 to 29) (Bastien, Fortier‐Brochu, et al. 2003; Vignola et al. 2000; Bang et al. 2022). Treatment duration was considerably shorter in the Mazza et al. (2014) study at 3.5 years (range: 2 to 6 years) compared to the remaining studies where mean duration ranged from 11.8 to 13.5 years (Bastien, Fortier‐Brochu, et al. 2003; Vignola et al. 2000; Bang et al. 2022).

Based on this overview of studies, a meta‐analysis was performed on BZD use in patients with insomnia based on the four studies. This was followed by an NMA combining study arms from Bastien, Fortier‐Brochu, et al. (2003), Vignola et al. (2000), and Bang et al. (2022).

The meta‐analysis based on the four studies showed a pooled BDI score for BZD insomniac patients of 9.30 (95% CI 7.96 to 10.64) with moderate statistical heterogeneity (*p* = 0.08, I^2^ = 56%) (Figure 5). When considering the network to get comparative measures (Figure 6A), patients with insomnia and treated with long‐term BZD showed a significantly higher BDI score compared to good sleepers (MD 6.84 (95% CI 5.15 to 8.53)) and a similar BDI score compared to drug‐free patients (0.30 (−1.49 to 2.10), Figure 6B).

**FIGURE 5:**
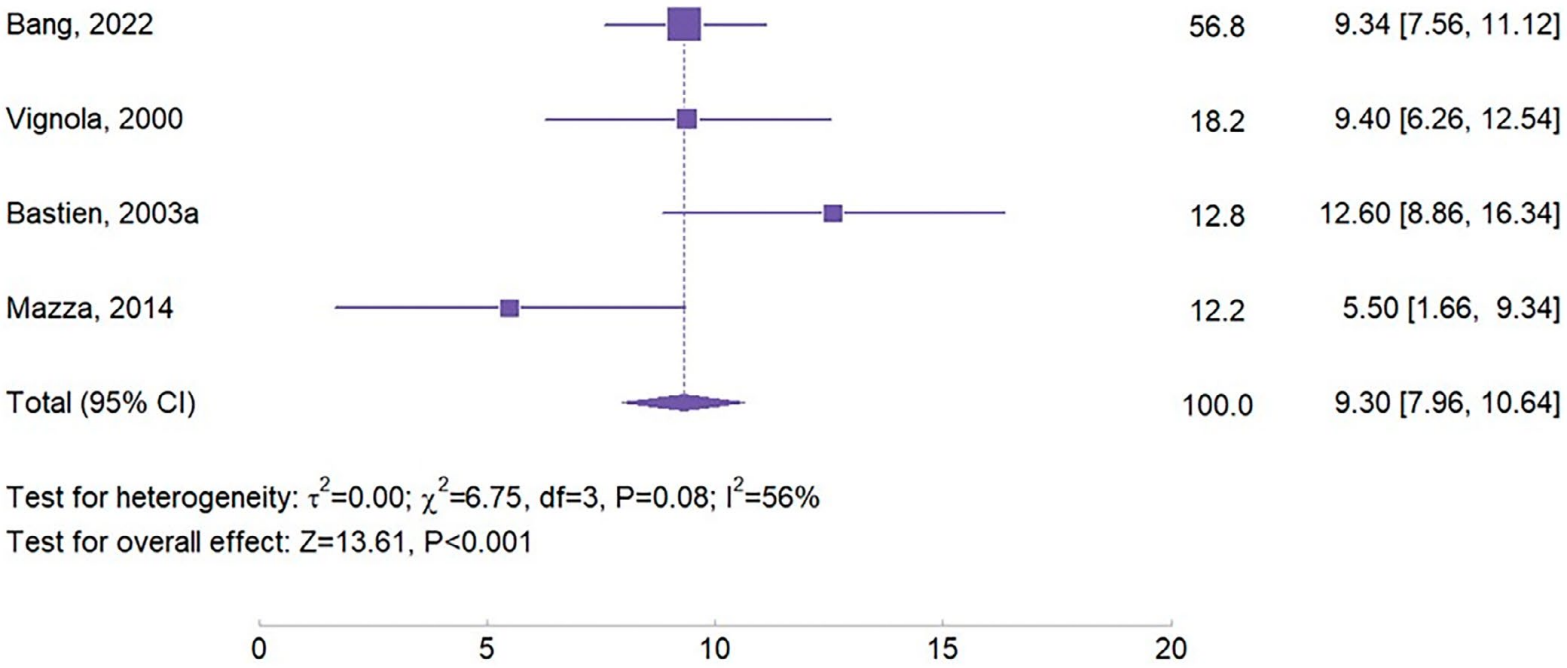
Meta‐analysis forest plot for BDI. BDI, beck depression inventory.

**FIGURE 6:**
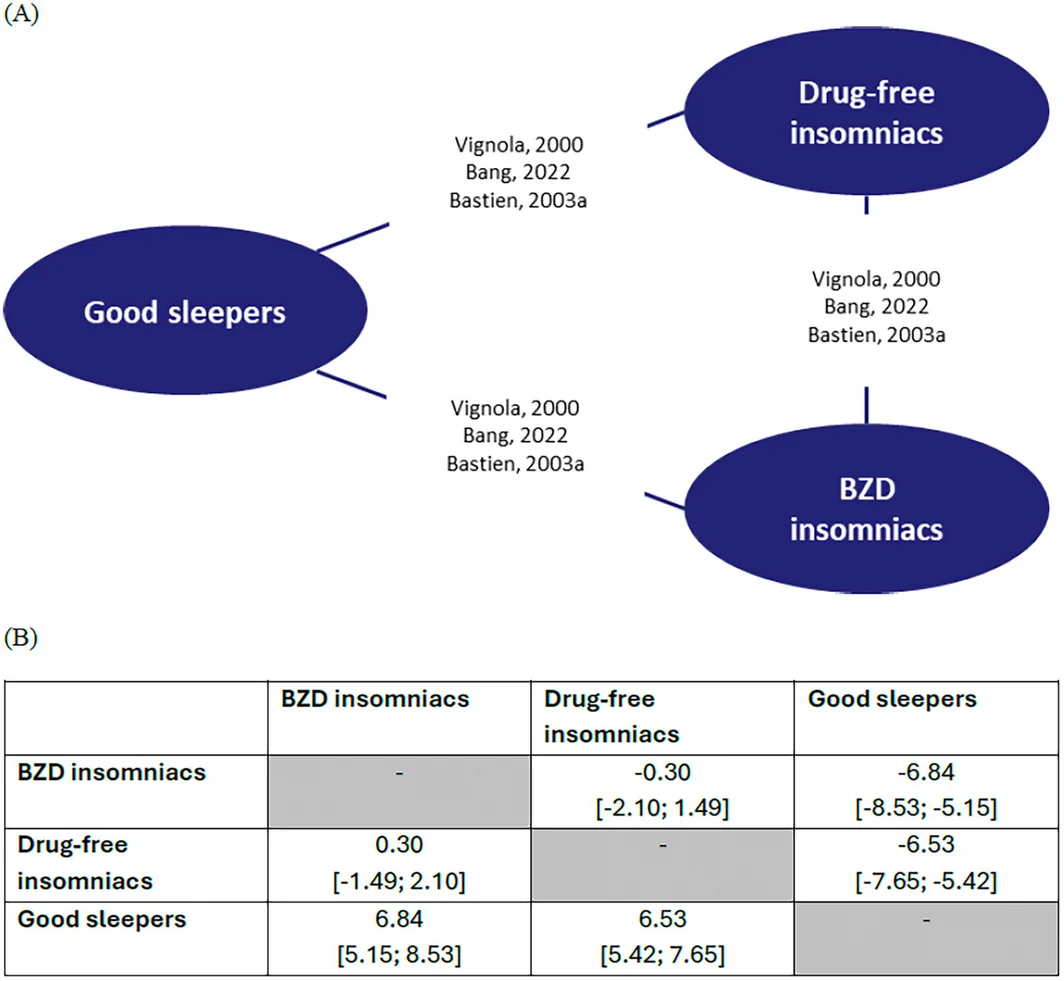
NMA network (A) and league table (B) for BDI. BDI, beck depression inventory.

##### 3.5.3.2 Anxiety

Two studies, Bastien, LeBlanc, et al. (2003) and Vignola et al. (2000), assessed anxiety in addition to depression in patients using the BAI. A feasibility assessment for a meta‐analysis was also conducted for BAI, with relevant study information presented in Table 3. Baseline characteristics were similar between the two studies in design (both prospective observational studies), geography (both from Canada), population (both assessing patients over 55 years of age), groups assessed (patients with chronic insomnia taking and not taking BZDs and a healthy control arm), and definition of insomnia (both using the DSM‐IV). Likewise, baseline characteristics were also consistent between the two studies, particularly in sample size, age, proportion of males, and years of education.

With these findings, a NMA leveraging the three groups per study was conducted and complemented with a meta‐analysis in which a pooled BAI mean score was obtained for patients with chronic insomnia and BZD use. Bastien, LeBlanc, et al. (2003) and Vignola et al. (2000) reported similar anxiety symptoms within insomnia patients using BZD groups (8.9 and 8.6, respectively) as well as drug‐free patients (7.5 and 9.1, respectively) and higher compared to good‐sleeper subjects (0.9 and 1.2, respectively). The NMA (Figure 7A) showed patients with insomnia taking BZD had significantly higher BAI scores compared to good sleepers (MD 7.53 (95% CI 5.35 to 9.71)) and a slightly numerically higher score compared to drug‐free patients (0.54 (−2.32 to 3.40), see Figure 7B), a higher BAI score indicating a higher degree of anxiety. The meta‐analysis performed on patients with insomnia using BZD groups showed a pooled BAI score of 8.67 (6.55 to 10.79) with no statistical heterogeneity (*p* = 0.90, I^2^ = 0%; see Figure 8).

**FIGURE 7:**
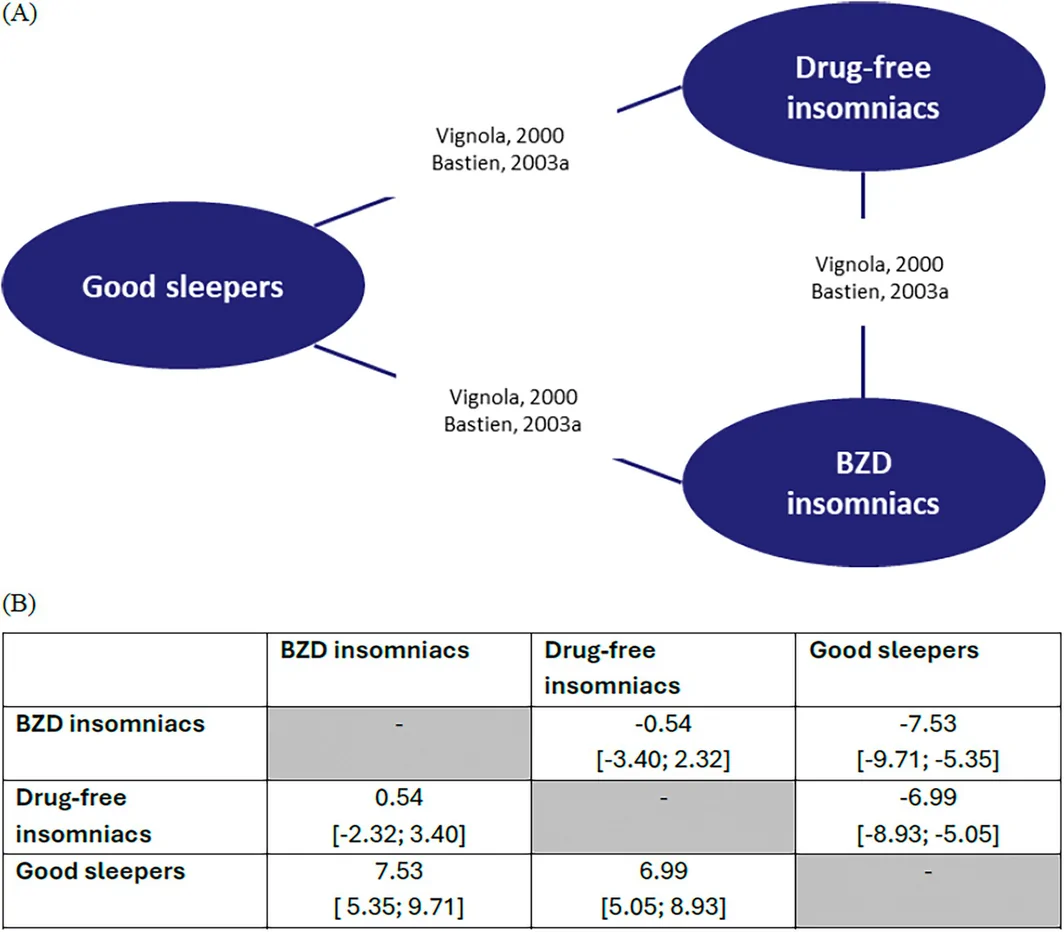
NMA network (A) and league table (B) for BAI. BAI, beck anxiety inventory; NMA, network meta‐analysis.

**FIGURE 8:**
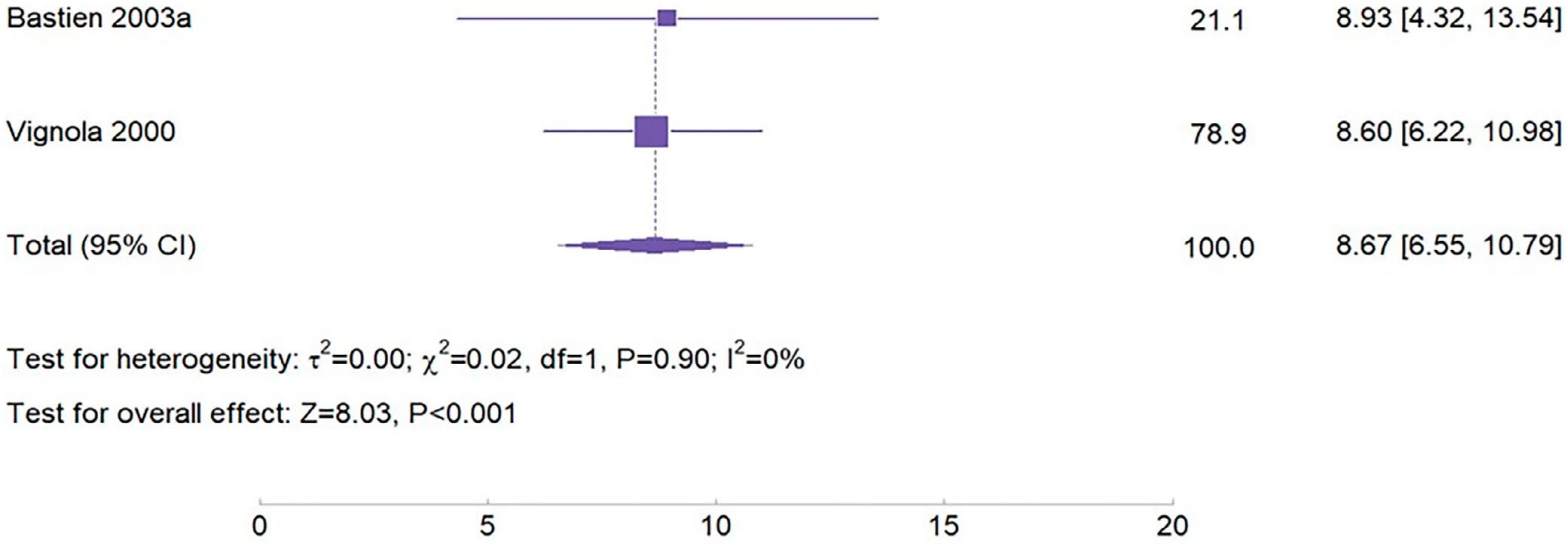
Meta‐analysis forest plot for BAI. BAI, beck anxiety inventory.

The narrative synthesis of findings in combination with the NMA results suggests increased prevalence of mental health morbidities in patients with insomnia who were BZD users compared to drug‐free patients and good sleepers.

### 3.6 Quality of Life Outcomes

Quality of life was reported in two studies (two publications) which were assessed through the 36‐Item Short Form Survey (SF‐36) questionnaire and perceived risk and benefit scores of BZD use (Supporting Information Table 13). Scores on the SF‐36 improved across all scales (e.g., physical functioning, mental health, general health, social functioning) in patients receiving JW‐SZRD in combination with lorazepam (treatment group) as well as those receiving lorazepam only (control group) (Song et al. 2020). Mah and Upshur (2002) reported significantly higher benefits of long‐term BZD use in patients themselves compared to physicians and, interestingly, also reported greater perceived risks compared to physicians. Unfortunately, neither study reported comparisons of outcome in BZD users to drug‐free patients or good sleepers (Mah and Upshur 2002; Song et al. 2020).

An overview of all outcomes assessed is provided in Supporting Information Figure 2 and includes the results of meta‐analyses and NMAs, where available, across sleep quality, safety, mental health, and quality of life outcomes.

## 4 Discussion

To our knowledge, this is the first systematic literature review centered on outcomes associated with long‐term benzodiazepine use (≥ 3 months) in patients with chronic insomnia. The review identified 28 publications from 27 studies (21 observational, 3 RCTs, and 3 nRCTs) published between 1987 and 2023, highlighting limitations due to the small number of relevant studies.

Although treatment guidelines recommend short‐term BZD use of up to 4 weeks, approximately one‐third of use in developed countries is long‐term, primarily among older adults (Olfson et al. 2015). This SLR and meta‐analysis assessed the impact of long‐term BZD use on sleep quality, safety, mental health, and QoL in patients with chronic insomnia. Despite the limited number of studies and their heterogenous study designs and outcome reporting, the studies consistently indicate poorer outcomes for patients with chronic insomnia using BZDs compared to those not using BZDs or other medications, or to good sleepers. Consequently, the findings support cautious short‐term BZD prescriptions, particularly when first‐line treatment (i.e., CBT‐I) is unavailable or unsuitable.

The literature on the effects of long‐term BZD use on sleep quality, particularly in patients with chronic insomnia, is limited. A meta‐analysis evaluating the effects of various sleep medications on both healthy adults and individuals with insomnia found that while BZD improve sleep parameters, their use should be restricted due to side effects (Wang et al. 2021). However, most alternative medications, such as H1‐antagonists and DORAs demonstrated superior effects on sleep quality compared to BZDs (Wang et al. 2021). While this SLR did not directly compare long‐term BZDs use to placebo, it assesses the association with chronic insomnia patients not using medication. Consistent with existing literature, BZD use was linked to lower severity of insomnia symptoms. However, studies focusing on older adults found that long‐term BZD use worsened sleep quality over time, both in individuals with baseline sleep difficulties and in good sleepers, albeit at a slower rate than the latter group (Béland et al. 2011). Poorer quality of sleep associated with BZD use in older adults has been assessed using the PSQI, where findings were consistent across all domains (i.e., subjective sleep quality, sleep latency, sleep duration, sleep efficacy, sleep disturbances, and daytime dysfunction). Similarly, this SLR found that long‐term BZD use was associated to overall poor sleep quality (pooled PSQI score > 5) although the included studies were highly statistically heterogenous (Buysse et al. 1989). Notably, a study of older adults indicated that faster‐acting BZDs were more strongly linked to decreased sleep quality than long‐acting BZDs, which may help explain inconsistencies in findings related to BZD use and sleep quality, warranting attention in future research (Chen et al. 2016).

Safety outcomes examined in this SLR, related to long‐term BZD use, particularly regarding alcohol dependence, abuse, or addiction, have been understudied (Griffiths and Weerts 1997; O'brien 2005).

The majority of the included studies showed BZD use to be associated with significant cognitive impairment compared to drug‐free patients and good sleepers. Evidence shows that in elderly patients, symptoms of drug‐induced cognitive disorders improved once the drug was discontinued (Gray et al. 1999). This risk may be specific to elderly populations, with long‐acting BZDs and prolonged duration (Chan et al. 2017; Billioti de Gage et al. 2014; AlDawsari et al. 2022). Our findings underscore the need for further research to pool results from studies of similar design.

Safety factors associated with long‐term BZD use include psychomotor impairment, increased risk of falls, injuries, traffic accidents, and mortality (Gray et al. 1999; Cumming and Conteur 2003). Falls risk was particularly noted in our findings, though a NMA found no significant difference in fall risk between long‐term BZD users and those on alternative medications, with only two studies included, suggesting the need for further research. Three studies in this SLR reported mortality outcomes, with one indicating increased mortality at higher BZD doses (Schonmann et al. 2018; Sato et al. 2020; Choi et al. 2018). While limited, the existing literature on mortality linked to long‐term BZD use in chronic insomnia generally supports our findings. Kripke et al. 2012, reported higher mortality rates in adults using temazepam for > 3 months, though not specifically in chronic insomnia patients. On the other hand, Patorno et al. (2017) did not find increased mortality rates with BZD use in adults based on a large population based study cohort not focusing on chronic insomnia. The sedative side effects contributing to psychomotor impairments may explain the increased mortality risk (Guilleminault 1990; Neutel 1995; Hwang et al. 2015).

Regarding mental health, this SLR found that long‐term use of BZDs in chronic insomnia was significantly associated with a higher prevalence of depression and anxiety compared to good sleepers, with little to no statistical heterogeneity. NMAs showed that depressive and anxious symptoms were significantly higher in patients taking BZDs compared to good sleepers but were similar to non‐medicated patients. Previous studies have reported higher anxiety and depression in BZD users (Manthey et al. 2011), with higher doses linked to worsened symptoms (Colizzi et al. 2021). The bidirectional relationship between insomnia and psychiatric comorbidities is well established (Taylor et al. 2007; Kalmbach et al. 2019) and improvement of insomnia with CBTi (Hertenstein et al. 2022) or Daridorexant has shown to subsequently improve anxiety and depression symptoms (Palagini et al. 2024). Our findings demonstrate that managing insomnia with BZD did not have a positive impact on depression and anxiety.

Although past SLRs and meta‐analyses of RCTs have reported on overall BZD side effects compared to other drugs and placebo (Crescenzo et al. 2022; Holbrook et al. 2000; Chiu et al. 2021), there is a lack of comprehensive reviews assessing the prevalence of specific outcomes (e.g., sleep quality and falls risk) associated with long‐term BZD use in chronic insomnia patients (Buscemi et al. 2007; Chiu et al. 2021).

This review has several strengths. It identified 28 publications from 27 studies, encompassing diverse study designs (21 observational studies, 3 RCTs, and 3 nRCTs), unlike many previous reviews focused exclusively on RCTs (Crescenzo et al. 2022; Holbrook et al. 2000; Chiu et al. 2021). The literature was retrieved from multiple databases, covering years 1987–2023 and considered multiple chronic insomnia criteria, allowing for a broad range of patients. This review also identified an extensive list of outcomes associated with long‐term BZD use in chronic insomnia.

However, the findings of this SLR and quantitative analyses are to be interpreted with caution due to limitations. The inclusion criteria were restricted to BZDs use of ≥ 3 months, missing studies on shorter durations that exceed the 4‐week guideline. BZDs may also have been prescribed for other indications (e.g., anxiety), affecting results. Next, the feasibility assessment for meta‐analyses and NMAs revealed the analysis was only feasible for five outcomes. This was in part due to our search yielding fewer studies of long‐term BZD use than expected along with the high heterogeneity across studies (e.g., study design, chronic insomnia definition). QoL was underreported, and there was insufficient data on patient characteristics (e.g., race, socio‐economic status) to allow for stratified analyses. The search focused solely on chronic insomnia, limiting the outcomes associated with BZD use for other conditions. Likewise, we conducted comparisons through meta‐analyses and NMAs based on broad categories (e.g., patients with chronic insomnia treated with any hypnotic other than a BZD), possibly reducing generalizability of results. Further, there was a lack of studies on the development of dependency linked to long‐term BZD use; however, this may be in part due to product label warnings that reduce the likelihood of dependency. Finally, it may often be the case that patients taking BZDs enrolled in observational trials experienced greater perceived symptoms and risks than those enrolled in and later prescribed BZDs in RCTs and nRCTs (i.e., patients found to have high depressive symptoms in observational studies may have had these symptoms prior to BZD use). Due to the much greater inclusion of observational studies compared to RCTs or nRCTs in the present review, the presence of risks in BZD users may have appeared higher than what was due to long‐term use of BZDs, and therefore differences between BZD and non‐BZD users may have appeared greater than they would have been in a controlled setting.

Further research is needed, particularly on outcomes related to long‐term BZDs use in chronic insomnia, including long‐term follow‐up RCTs to support non‐randomized studies. Larger studies should assess the safety of BZDs use for chronic insomnia in high‐risk groups, such as the elderly, and better characterize BZD abuse risk, especially in older populations. As suggested by only five outcomes being quantitatively analysed after a feasibility assessment of studies, future studies should aim for consistency in study design, population characteristics, including duration and definition of chronic insomnia, timepoints of outcome assessment, and outcome assessment methods.

Additionally, more data comparing short‐term and long‐term BZDs use are needed. European guidelines recommend pharmacological treatments only if CBT‐I is ineffective (Palagini et al. 2023). Efforts should focus on increasing access to first‐line insomnia treatments, promoting CBT‐I, and integrating CBT‐I experts into standard care worldwide (Baglioni et al. 2020; Baglioni et al. 2023; Espie and Henry 2023; Schneider et al. 2023; Schneider et al. 2020). In the meanwhile, a balance should also be found between a delivery by appropriate experts and facilitating the access to the best intervention (Baglioni et al. 2023; Takaesu et al. 2019). Pharmacotherapy is used when CBT‐I is ineffective but the use of BZD should be in general limited to 4 weeks. The European guidelines, however, recommend the use of DORAs over GABA agonists (i.e., BZDs) for 3 months or longer, if needed, largely due to their differing mechanisms of action (Riemann et al. 2023). It is believed that the binding and activation of the neuropeptides Orexin A and Orexin B to Orexin Receptor type 1 and Orexin Receptor type 2 contribute to the disruption of sleep onset and sleep maintenance, respectively (Roch et al. 2021). DORAs work by blocking this pathway, thereby promoting sleep and helping to maintain a healthy sleep cycle (Roch et al. 2021; Robinson et al. 2022). Further, the more targeted activity of DORAs, compared to the widespread effects of GABA antagonists, lend to reduced adverse events with DORA use, such as those described in the current SLR, compared to GABA antagonist use (Sarathi Chakraborty et al. 2023). Added to this, use of DORAs have been linked to low likelihood for abuse or for tolerance when used on the long term (Roch et al. 2021).

In conclusion, this SLR and meta‐analysis is the first to examine long‐term BZDs use (≥ 3 months) in patients with chronic insomnia. Our findings suggest negative impacts on sleep quality, safety, mental health, and QoL, particularly in insomnia symptom severity, and depressive and anxious symptoms when compared to good sleepers. These effects were generally worse compared to good sleepers, though results compared to non‐BZD were less clear. Although there were limitations, this SLR provides a base upon which future reviews may expand. Despite limitations, this review provides a foundation for future research. We recommend restricting BZD use to the short‐term (maximum of 4 weeks) and promoting CBT‐I, with safer pharmacotherapies considered when necessary.

## Author Contributions

**Dieter Riemann:** conceptualization, writing – review and editing. **Chiara Baglioni:** conceptualization, writing – review and editing. **Christoph Nissen:** conceptualization, writing – review and editing. **Agathe Nevière:** conceptualization, formal analysis, methodology, project administration, writing – original draft, writing – review and editing. **Omar Irfan:** conceptualization, formal analysis, investigation, writing – original draft, writing – review and editing. **Pauline Le Nouveau:** conceptualization, supervision, validation, project administration, writing – review and editing, writing – original draft. **Chrysoula Leontiou:** conceptualization, supervision, writing – original draft, writing – review and editing. **Damien Léger:** conceptualization, writing – review and editing.

## Funding

This work was supported by Idorsia Pharmaceuticals Ltd. Idorsia played an active role in the study design and in the interpretation of the study results.

## Ethics Statement

As this study was a systematic review and meta‐analysis of previously published studies, ethics approval was not required.

## Conflicts of Interest

Pr Rieman is member of Speaker's panel and advisory board: Idorsia, HelloBetter; Member of Advisory board: Xtrodes, Gaia AG Pr Nissen has served on advisory boards of Idorsia, Janssen and the GetOn Institute. Chrysoula Leontiou is an employee of Idorsia pharmaceuticals. Agathe Nevière, Omar Irfan and Pauline Le Nouveau: Employees of Amaris Consulting. Pr Leger has been granted in the las 5 years for himself or its institution by Jazz, Linda, Idorsia, Bioserenity, Vitalaire, Sanofi, iSommeil, Agence innovation défense, LVMH research. All authors declare no COI on this manuscript.

## Supporting information


**Supporting Information: Table 1.** Search terms and results from EMBASE and MEDLINE for 2012–2022.
**Supporting Information: Table 2** Search terms and results from EMBASE and MEDLINE for 1987–2011.
**Supporting Information: Table 3** Search terms and results from MEDLINE‐in‐process for 2012–2022.
**Supporting Information: Table 4** Search terms and results from MEDLINE‐in‐process for 1987–2011.
**Supporting Information: Table 5** Search terms and results from Cochrane CENTRAL for 2012–2022.
**Supporting Information: Table 6** Search terms and results from Cochrane CENTRAL for 1987–2011.
**Supporting Information: Table 7** Search terms and results from PsycInfo for 2012–2022.
**Supporting Information: Table 8** Search terms and results from PsycInfo for 1987–2011.
**Supporting Information: Table 9** Baseline characteristics of patients with chronic insomnia from studies included in the SLR (27 studies, 28 publications).
**Supporting Information: Table 10** Outcomes reported in studies included in the SLR assessing sleep quality (9 studies, 10 publications).
**Supporting Information: Table 11** Outcomes reported in studies included in the SLR assessing safety outcomes (21 studies, 22 publications).
**Supporting Information: Table 12** Outcomes reported in studies included in the SLR assessing mental health outcomes (9 studies, 10 publications).
**Supporting Information: Table 13** Outcomes reported in studies included in the SLR assessing QoL outcomes (2 studies, 2 publications).
**Supporting Information: Figure 1** PRISMA flow diagram of the overall SLR process detailed for the two searches (Original SLR: 2012–2023, Updated SLR: 1987–2011).
**Supporting Information: Figure 2** Overview of outcomes assessed by MA and NMA.

## Data Availability

Data files and statistical codes are available by request to the corresponding author.
