# Supplementary material for: Risks Associated With Benzodiazepine Long‐Term Use in Chronic Insomnia: A Systematic Review and (Network) Meta‐Analysis
Source: J Sleep Res. 2026 Jun 11;35(5):e70352. doi: 10.1111/jsr.70352 (PMC13619061; doi:10.1111/jsr.70352)
Supplement: Supplementary file 1 [file JSR-35-e70352-s001.docx]

**Risks associated with benzodiazepine long-term use in chronic insomnia: A systematic review and (network) meta-analysis**

# Supplemental Material

**Supplemental Tables**

[Supplemental Table 1. Search terms and results from EMBASE and MEDLINE for 2012-2022	2](#_Toc191049316)

[Supplemental Table 2. Search terms and results from EMBASE and MEDLINE for 1987-2011	4](#_Toc191049317)

[Supplemental Table 3. Search terms and results from MEDLINE-in-process for 2012-2022	6](#_Toc191049318)

[Supplemental Table 4. Search terms and results from MEDLINE-in-process for 1987-2011	7](#_Toc191049319)

[Supplemental Table 5. Search terms and results from Cochrane CENTRAL for 2012-2022	8](#_Toc191049320)

[Supplemental Table 6. Search terms and results from Cochrane CENTRAL for 1987-2011	9](#_Toc191049321)

[Supplemental Table 7. Search terms and results from PsycInfo for 2012-2022	10](#_Toc191049322)

[Supplemental Table 8. Search terms and results from PsycInfo for 1987-2011	11](#_Toc191049323)

[Supplemental Table 9. PICOS Table outlining inclusion and exclusion criteria for the SLR search	12](#_Toc191049324)

[Supplemental Table 10. Baseline characteristics of patients with chronic insomnia from studies included in the SLR (27 studies, 28 publications)	14](#_Toc191049325)

[Supplemental Table 11. Baseline characteristics of studies included in the SLR (27 studies, 28 publications)	18](#_Toc191049326)

[Supplemental Table 12. Outcomes reported in studies included in the SLR assessing sleep quality (9 studies, 10 publications)	22](#_Toc191049327)

[Supplemental Table 13. Overview and baseline characteristics of studies included in feasibility assessment for potential meta-analyses and NMAs (10 studies)	24](#_Toc191049328)

[Supplemental Table 14. Outcomes reported in studies included in the SLR assessing safety outcomes (21 studies, 22 publications)	28](#_Toc191049329)

[Supplemental Table 15. Outcomes reported in studies included in the SLR assessing mental health outcomes (9 studies, 10 publications)	31](#_Toc191049330)

[Supplemental Table 16. Outcomes reported in studies included in the SLR assessing QoL outcomes (2 studies, 2 publications)	33](#_Toc191049331)

**Supplemental Figures**

[Supplemental Figure 1. PRISMA flow diagram of the overall SLR process detailed for the two searches (Original SLR: 2012-2023, Updated SLR: 1987-2011)	34](#_Toc191049247)

[Supplemental Figure 2. Overview of outcomes assessed by MA and NMA	35](#_Toc191049248)

Supplemental Table 1. Search terms and results from EMBASE and MEDLINE for 2012-2022

| No | Query | Results |
| --- | --- | --- |
| #1 | 'chronic insomnia'/de OR 'agrypnia' OR 'hyposomnia' OR 'sleep initiation and maintenance disorders' OR 'sleeplessness' OR 'early awakening':ti,ab OR 'chronic insomni*':ti,ab OR 'nocturnal awakening':ti,ab OR 'sleep onset':ti,ab | 15012 |
| #2 | 'clinical trial'/de | 1063176 |
| #3 | 'randomized controlled trial'/de | 739786 |
| #4 | 'controlled clinical trial'/de | 438561 |
| #5 | 'multicenter study'/de | 341912 |
| #6 | 'phase 3 clinical trial'/de | 64094 |
| #7 | 'phase 4 clinical trial'/de | 5049 |
| #8 | 'randomization'/exp | 95829 |
| #9 | 'single blind procedure'/de | 48441 |
| #10 | 'double blind procedure'/de | 201645 |
| #11 | 'crossover procedure'/de | 72145 |
| #12 | 'placebo'/de | 396255 |
| #13 | 'randomi?ed controlled trial$':ti,ab | 299716 |
| #14 | rct:ti,ab | 50051 |
| #15 | (random$ NEAR/2 allocat$):ti,ab | 3 |
| #16 | 'single blind$':ti,ab | 21392 |
| #17 | 'double blind$':ti,ab | 217700 |
| #18 | ((treble OR triple) NEAR/1 blind$):ti,ab | 1408 |
| #19 | placebo$:ti,ab | 352249 |
| #20 | 'prospective study'/de | 811110 |
| #21 | #2 OR #3 OR #4 OR #5 OR #6 OR #7 OR #8 OR #9 OR #10 OR #11 OR #12 OR #13 OR #14 OR #15 OR #16 OR #17 OR #18 OR #19 OR #20 | 2787936 |
| #22 | 'case study'/de | 89386 |
| #23 | 'case report':ti,ab | 520543 |
| #24 | 'abstract report'/de OR 'letter'/de | 1265590 |
| #25 | 'conference proceeding':it | 0 |
| #26 | 'conference abstract':it | 4604231 |
| #27 | editorial:it | 740546 |
| #28 | letter:it | 1240113 |
| #29 | note:it | 908171 |
| #30 | #22 OR #23 OR #24 OR #25 OR #26 OR #27 OR #28 OR #29 | 8042711 |
| #31 | #21 NOT #30 | 2014357 |
| #32 | 'clinical study'/de | 161147 |
| #33 | 'case control study' | 244285 |
| #34 | 'family study'/de | 26239 |
| #35 | 'longitudinal study'/de | 181723 |
| #36 | 'retrospective study'/de | 1344047 |
| #37 | 'prospective study'/de | 811110 |
| #38 | 'randomized controlled trials'/de | 239499 |
| #39 | #37 NOT #38 | 801478 |
| #40 | 'cohort analysis'/de | 921164 |
| #41 | (cohort NEAR/1 (study OR studies)):ti,ab,kw | 455946 |
| #42 | ('case control' NEAR/1 (study OR studies)):ti,ab | 159990 |
| #43 | ('follow up' NEAR/1 (study OR studies)):ti,ab | 76741 |
| #44 | (observational NEAR/1 (study OR studies)):ti,ab | 231243 |
| #45 | (epidemiologic$ NEAR/1 (study OR studies)):ti,ab | 35214 |
| #46 | ('cross sectional' NEAR/1 (study OR studies)):ti,ab | 309903 |
| #47 | #32 OR #33 OR #34 OR #35 OR #36 OR #39 OR #40 OR #41 OR #42 OR #43 OR #44 OR #45 OR #46 | 3587072 |
| #48 | #31 OR #47 | 4897906 |
| #49 | #1 AND #48 | 4161 |
| #50 | #49 AND ('Article'/it OR 'Article in Press'/it) | 2878 |
| #51 | #49 AND ('article'/it OR 'article in press'/it) AND [humans]/lim AND [english]/lim | 2821 |
| #52 | #49 AND ('article'/it OR 'article in press'/it) AND [humans]/lim AND [english]/lim AND [2012-2022]/py | 1746 |

**Note**: Search was conducted on 21 November 2022.

Supplemental Table 2. Search terms and results from EMBASE and MEDLINE for 1987-2011

| No | Query | Results |
| --- | --- | --- |
| #1 | 'chronic insomnia'/de OR 'agrypnia' OR 'hyposomnia' OR 'sleep initiation and maintenance disorders' OR 'sleeplessness' OR 'early awakening':ti,ab OR 'chronic insomni*':ti,ab OR 'nocturnal awakening':ti,ab OR 'sleep onset':ti,ab | 15181 |
| #2 | 'clinical trial'/de | 1066634 |
| #3 | 'randomized controlled trial'/de | 748102 |
| #4 | 'controlled clinical trial'/de | 438998 |
| #5 | 'multicenter study'/de | 347504 |
| #6 | 'phase 3 clinical trial'/de | 65046 |
| #7 | 'phase 4 clinical trial'/de | 5125 |
| #8 | 'randomization'/exp | 96264 |
| #9 | 'single blind procedure'/de | 49034 |
| #10 | 'double blind procedure'/de | 203426 |
| #11 | 'crossover procedure'/de | 72698 |
| #12 | 'placebo'/de | 398795 |
| #13 | 'randomi?ed controlled trial$':ti,ab | 304353 |
| #14 | rct:ti,ab | 50987 |
| #15 | (random$ NEAR/2 allocat$):ti,ab | 3 |
| #16 | 'single blind$':ti,ab | 21609 |
| #17 | 'double blind$':ti,ab | 219096 |
| #18 | ((treble OR triple) NEAR/1 blind$):ti,ab | 1436 |
| #19 | placebo$:ti,ab | 355124 |
| #20 | 'prospective study'/de | 823989 |
| #21 | #2 OR #3 OR #4 OR #5 OR #6 OR #7 OR #8 OR #9 OR #10 OR #11 OR #12 OR #13 OR #14 OR #15 OR #16 OR #17 OR #18 OR #19 OR #20 | 2816860 |
| #22 | 'case study'/de | 90577 |
| #23 | 'case report':ti,ab | 526864 |
| #24 | 'abstract report'/de OR 'letter'/de | 1271569 |
| #25 | 'conference proceeding':it | 0 |
| #26 | 'conference abstract':it | 4665862 |
| #27 | editorial:it | 745931 |
| #28 | letter:it | 1246799 |
| #29 | note:it | 914935 |
| #30 | #22 OR #23 OR #24 OR #25 OR #26 OR #27 OR #28 OR #29 | 8127068 |
| #31 | #21 NOT #30 | 2030546 |
| #32 | 'clinical study'/de | 161628 |
| #33 | 'case control study' | 246518 |
| #34 | 'family study'/de | 26260 |
| #35 | 'longitudinal study'/de | 184670 |
| #36 | 'retrospective study'/de | 1370641 |
| #37 | 'prospective study'/de | 823989 |
| #38 | 'randomized controlled trials'/de | 243507 |
| #39 | #37 NOT #38 | 814154 |
| #40 | 'cohort analysis'/de | 944366 |
| #41 | (cohort NEAR/1 (study OR studies)):ti,ab,kw | 465208 |
| #42 | ('case control' NEAR/1 (study OR studies)):ti,ab | 161629 |
| #43 | ('follow up' NEAR/1 (study OR studies)):ti,ab | 77380 |
| #44 | (observational NEAR/1 (study OR studies)):ti,ab | 236187 |
| #45 | (epidemiologic$ NEAR/1 (study OR studies)):ti,ab | 35359 |
| #46 | ('cross sectional' NEAR/1 (study OR studies)):ti,ab | 316769 |
| #47 | #32 OR #33 OR #34 OR #35 OR #36 OR #39 OR #40 OR #41 OR #42 OR #43 OR #44 OR #45 OR #46 | 3647100 |
| #48 | #31 OR #47 | 4964880 |
| #49 | #1 AND #48 | 4222 |
| #50 | #49 AND ('Article'/it OR 'Article in Press'/it) | 2918 |
| #51 | #49 AND ('article'/it OR 'article in press'/it) AND [humans]/lim AND [english]/lim | 2738 |
| #52 | #49 AND ('article'/it OR 'article in press'/it) AND [humans]/lim AND [english]/lim AND [1987-2011]/py | **862** |

**Note**: Search was conducted on 18 January 2023.

Supplemental Table 3. Search terms and results from MEDLINE-in-process for 2012-2022

| No. | Query | Results |
| --- | --- | --- |
| #1 | “chronic insomnia” OR “chronic insomni*” OR insomni* OR "sleep disorder*" OR "sleep wake disorder*" OR "sleep initiation" OR "sleep onset" OR "sleep latency" | 82,903 |
| #2 | #1 AND human AND English | 63,296 |
| #3 | #2 AND (2012-2022) | 36,229 |
| #4 | #3 AND (inprocess[sb] OR pubstatusaheadofprint) | 39 |

**Note**: Search was conducted on 21 November 2022.

Supplemental Table 4. Search terms and results from MEDLINE-in-process for 1987-2011

| No. | Query | Results |
| --- | --- | --- |
| #1 | “chronic insomnia” OR “chronic insomni*” OR insomni* OR "sleep disorder*" OR "sleep wake disorder*" OR "sleep initiation" OR "sleep onset" OR "sleep latency" | 83,944 |
| #2 | #1 AND human AND English | 64012 |
| #3 | #2 AND (1987-2011) | 23,707 |
| #4 | #3 AND (inprocess[sb] OR pubstatusaheadofprint) | **0** |

**Note**: Search was conducted on 18 January 2023.

Supplemental Table 5. Search terms and results from Cochrane CENTRAL for 2012-2022

| No. | Query | Results |
| --- | --- | --- |
| #1 | 'chronic insomnia' OR 'chronic insomni*' | 2733 |
| #2 | #1 with Publication Year from 2012 to 2022, in Trials and Cochrane Protocol | 1588 |

**Note**: Search was conducted on 21 November 2022.

Supplemental Table 6. Search terms and results from Cochrane CENTRAL for 1987-2011

| No. | Query | Results |
| --- | --- | --- |
| #1 | 'chronic insomnia' OR 'chronic insomni*' | 2762 |
| #2 | #1 with Publication Year from 1987 to 2011, in Trials and Cochrane Protocol | 651 |

**Note**: Search was conducted on 18 January 2023.

Supplemental Table 7. Search terms and results from PsycInfo for 2012-2022

| No. | Query | Results |
| --- | --- | --- |
| #1 | (insomnia AND benzodiazepine) with Publication Year from 2012 to 2023 | 208 |

**Note**: Search was conducted on 4 January 2023.

Supplemental Table 8. Search terms and results from PsycInfo for 1987-2011

| No. | Query | Results |
| --- | --- | --- |
| #1 | (insomnia AND benzodiazepine) with Publication Year from 2012 to 2023 | 294 |

**Note**: Search was conducted on 24 January 2023.

Supplemental Table 9. Baseline characteristics of patients with chronic insomnia from studies included in the SLR (27 studies, 28 publications)

| Study | Treatment | Sample size, n | Male, % | Age, years  mean (SD) | Time since diagnosis | Duration of BZD use, mean (SD) | Sleep quality |
| --- | --- | --- | --- | --- | --- | --- | --- |
| Baillargeon et al., 2003 | Tapering BZD alone | 30 | 43.0 | 66.4 (6.0) | BZD arm: 277 months (SD=188) (mean) | 152 months | NR |
| Bang et al., 2022 | BZD insomnia patients | 29 | 34.5 | 68.0 (8.38) | >1 year | Median 11.8 years | ISI mean 11.66 (7.13) |
| Bastien et al., 2003a | BZD insomnia patients | 15 | 46.7 | 62.2 (6.0) | BZD arm: 17.5 years (SD=8.3) (mean) | 13.5 years (SD=10) | ISI mean 16.5 (4.4) |
| Chen et al., 2012 | BZD insomnia patients | 1,915 | NR | NR | Long-term insomnia, diagnosed with insomnia twice within one year | NR | NR |
| Choi et al., 2018 | BZD insomnia patients | 1,954 | NR | NR | ≥2 claims of diagnosis/year | Followed for 11 years | NR |
| Chung et al., 2015 | Insomnia-hypnotic group (BZD + non-BZD) | 2,295 insomnia patients using BZD and non-BZD | 48.8 | NR | ≥1 month  With ≥2 claims of diagnosis/year | NR | NR |
| Declerck and Smits, 1999 | BZD insomnia patients | 22 | 29.0 | 54 | NR | 51 months | NR |
| Eto et al., 1998 | BZD insomnia patients | 25 | 32.0 | 77.9 (5.8) | Taking BZDs as hypnotics mostly every night for >1 month | 44.6 months (range 1.4 to 177.1) | NR |
| Fang et al., 2019 | BZD insomnia patients | 840 | NR | NR | ≥2 claims of diagnosis/year | NR | NR |
| Guo et al., 2021 | BZD insomnia patients | 30 | 20.8 | 64.2 (6.98) | 0.5-50 years | Mean 8.92 (SD=12.39) years* | ISI: mean 14.27 (SD=3.05) |
| Jiang et al., 2019 | BZD insomnia patients | 19 | NR | NR | Prospective follow-up for 1 year | NR | NR |
| Kudoh et al., 2004 | BZD insomnia patients | 31 | NR | NR | Long-term daily BZD use for >1 year | NR | NR |
| Lavigne et al., 2019 | BZD insomnia patients | 83,795 | 91.1 | 61.7 (15.6) | Received treatment for insomnia for 2 years | NR | NR |
| Mah and Upshur, 2002 | BZD insomnia patients | 93 | 33.0 | 77 | BZD prescriptions for >1 year duration for insomnia | NR | NR |
| Mazza et al., 2014 | BZD insomnia patients | 6 | 66.7 | 53.3 (14.8) | 2-6 years | 3.5 (2-6) years | PSQI: mean 9.7 (SD=4.1)  ESS: mean 3.7 (SD=4.3) |
| Miano et al., 2020** | BZD insomnia patients | 17 | NR | 49.2 (11.2) | NR | NR | NR |
| Poyares et al., 2004 | BZD insomnia patients | 25 | 16.0 | 44.3 (11.8) | ≥1 month | 6.8 (SD=5.4) years (range 2.2-14) | NR |
| Sato et al., 2020 | BZD insomnia patients | 488 | 55.9 | 72 (range 62-79) | NR | Followed up for 1254 days (3.4 years) | NR |
| Schenck and Mahowald, 1996 | BZD insomnia patients | 25 | 36.0 | 41.9 (13.9) | Treated for ≥6 months with nightly BZD | 4 (SD=2.9) years | NR |
| Schneider-Helmert et al., 1988 | BZD insomnia patients | 40 | 70.0 | NR | ≥6 months nightly BZD use | NR | NR |
| Schonmann et al., 2018 | BZD insomnia patients | 154,929 | 42.0 | 64.5 (16.3) | Long-term use of hypnotics | NR | NR |
| Schredl et al., 1998 | BZD insomnia patients | 52 | 48.1 | 50.2 (18.3) | NR | NR | NR |
| Shapiro et al., 1995 | Insomnia patients taking BZDs and then switched to Zopiclone | 134 | 45.5 | 49.9 (range 25-86) | Taking hypnotics between 3 months to 19 years | 3.1 years | NR |
| Shash et al., 2016 | BZD insomnia patients | 374 | NR | NR | NR | Association of BZD use and dementia over 8 years | NR |
| Song et al., 2020 | BZD insomnia patients | 94 | 33.0 | 39.3 (9.4) | ≥3 months | NR | NR |
| Primary study:  Vignola et al., 2000;  Secondary study:  Bastien et al., 2003b | BZD insomnia patients | 20 | 45.0 | 62.3 (5.9) | 25.8 years (SD=15.7) (mean) for total insomnia patients | 13.5 (SD= 10) | Sleep impairment index mean 16.85 (SD=4.55) |
| Yen et al., 2015 | BZD insomnia patients | 88 | NR | NR | ≥3 months | 115.6 months (hypnotic use) | NR |

*Average duration of sedative-hypnotics, including both BZD and Z-drugs; **This study was an abstract.

**Abbreviations**: BZD: Benzodiazepine; CBT: Cognitive behavioral therapy; ESS: Epworth Sleepiness Scale; ISI: Insomnia severity index; NR: Not reported; PSQI: Pittsburgh Sleep Quality Index; SD: Standard deviation; SLR: systematic literature review.

Supplemental Table . Outcomes reported in studies included in the SLR assessing sleep quality (9 studies, 10 publications)

| Study | Measures assessed | Outcomes |
| --- | --- | --- |
| Bang et al., 2022 | ISI  ESS | Significant differences in sleep quality were reported on the ISI, demonstrating that drug-free insomniacs had the highest score (mean 18.11 [SD 5.13]), representing worst sleep quality, followed by insomniacs using BZD (11.66 [7.13]), and the control group (4.89 [4.98]).  Differences were not seen between groups on the ESS (p=0.091 across groups). |
| Bastien et al., 2003a | ISI | Both the drug-free insomnia patients and insomnia patients with BZD use had significantly higher scores on the ISI (mean 18.5 [SD 4.9] and 16.5 [4.4], respectively) compared to the good sleepers group (1.69 [1.9]; p<0.01). |
| Declerck and Smits, 1999 | Sleep latency time  Total sleep time  WASO | None of sleep latency time, total sleep duration, or WASO changed significantly after 14 days, in patients who switched from BDZ to zolpidem use, when compared to those who remained on a BZD. |
| Guo et al., 2021 | PSQI | In patients with chronic insomnia categorized by presence or absence of cognitive impairment, measured in part by object cognitive assessments, PSQI scores were similar between the two groups (p=0.87).  As well, when comparing PSQI scores based on BZD use, there were no significant differences found between groups (p=0.54). |
| Mazza et al., 2014 | Sleep latency time  Time in bed  Total sleep time  Sleep period time  Sleep efficiency  PSQI  ESS | Patients with a history of BZD abuse had short sleep onset latency compared to controls (p=0.04); however, no differences were found between the two group in time in bed, total sleep time, sleep period time, and sleep efficiency index (p>0.05 for all comparisons).  Of note, per-participant scores were provided for the PSQI and ESS. |
| Poyares et al., 2004 | Sleep latency time  Total sleep time  Sleep efficiency | Sleep latency, total sleep time, and sleep efficiency were significantly poorer in patients with long-term BZD use compared to controls (p=0.02, p=0.006, and p=0.007, respectively). |
| Schneider-Helmert et al., 1988 | Total sleep time  Sleep onset latency  WASO | No significant differences were found between patients with chronic insomnia taking vs. not taking medication when comparing total sleep time, sleep onset latency, or WASO. |
| Song et al., 2020 | Sleep latency time  Total sleep time  Number of nighttime awakenings | Patients receiving JW-SZRD and lorazepam had improved sleep onset latency, total sleep time, and number of nighttime awakenings over a 12-week period (p<0.001 for all trends) while the control group did not report the same (p>0.05 for all trends). |
| Primary study:  Vignola et al., 2000;  Secondary study:  Bastien et al., 2003b | Sleep latency time  WASO  Early morning awakenings  Total wake time  Total sleep time  Time in bed  Sleep efficiency | When compared to good sleepers, patients with chronic insomnia both taking and not taking BZD had significantly poorer sleep onset latency, wake after sleep onset, early morning awakening, total wake time, total sleep time, time in bed, and sleep efficiency (p<0.01 for all comparisons). |

**Abbreviations**: BZD: benzodiazepine; ESS: Epworth sleepiness scale; ISI: Insomnia severity index; JW-SZRD: Jiawei Suanzaoren Decoction; PSQI: Pittsburg Sleep Quality Index; SD: standard deviation; WASO: wake time after sleep onset.

Supplemental Table . Outcomes reported in studies included in the SLR assessing safety outcomes (21 studies, 22 publications)

| Study | Measures assessed | Outcomes |
| --- | --- | --- |
| Baillargeon et al., 2003 | Dependance | Higher odds of dependance were reported in the group undergoing tapering along compared to tapering combined with CBT (i.e., combined treatment).  At 12 months, the combined treatment group was more likely to be BZD-free (aOR 7.6, 95% CI: 2.5-26.6) and to have a dose reduction of at least 50% (aOR 4.1, 95% CI: 1.3-13.8), with adjustment for initial BZD dose. |
| Bang et al., 2022 | Cognition | Performance on measures of global cognition, attention, memory, executive function, and visuospatial abilities were lowest in the insomniacs using BZD group compared to the drug-free insomniacs and control groups.  These differences, however, were only significant on tests of executive function, namely on the TMT-A (p<0.001) and category fluency test (p=0.002).  Tests of memory were not significantly different between groups. |
| Chen et al., 2012 | Dementia risk | Dementia risk was higher in patients with a history of hypnotic usage compared to those without (HR 2.34, 95% CI : 1.92-2.85 ; p<0.001). |
| Choi et al., 2018 | Mortality | A significantly higher risk of death was reported in individuals with hypnotic use compared to those without (HR 1.14, 95% CI: 1.12-1.16). |
| Eto et al., 1998 | Falls  Gait and balance | An examination of gait and balance in BZD users and non-BZD users found no significant difference on balance metrics or on gait speed but did find that step length of BZD users was shorter than that of non-BZD users (p=0.04). |
| Fang et al., 2019 | Cancer risk | Compared to patients without insomnia (and not taking medication), those with insomnia using sedative-hypnotics had an increased risk of cancer (HR 1.49), as did those with insomnia not taking a sedative-hypnotic (HR 1.68). |
| Guo et al., 2021 | Cognition | In patients with chronic insomnia categorized by presence or absence of cognitive impairment, measured in part by object cognitive assessments, there was no difference in the proportion of BZD users between the two groups (p=0.07).  However, when comparing patients based on BZD use, those taking BZDs had lower global cognition scores (MMSE and MoCA) and a lower DST score. There were no differences reported on verbal memory (AVLT), executive function (TMT-B), and language (BNT-30). |
| Jiang et al., 2019 | Falls | Patients taking BZD were significantly more likely than non-BZD users to experience falls (p=0.03) and have injurious falls (p<0.001). |
| Kudoh et al., 2004 | Post-operative confusion (cognition)  Depression  Anxiety | Incidence of post-operative confusion after orthopedic surgery was significantly higher in patients taking BZD (26%) compared to non-BZD users (13%, p<0.05).  However, no differences were reported on that STAI or other depression scores. |
| Lavigne et al., 2019 | Alcohol use disorder  Substance use disorder | Rates of alcohol use disorder in individuals taking BZD were not found to be higher than other groups assessed in the study (4.5% compared to 4.0% of zolpidem users, 9.1% of trazodone users, and 7.9% of antihistamine users).  Similar findings were reported on rates of substance use disorder (2.3% vs. 2.1%, 5.7%, and 5.7%, respectively). |
| Miano et al., 2020* | Cognition | Chronic insomnia patients with reported history of BZD abuse performed significantly worse on tests of global cognition (MMSE) and executive function (semantic and phonetic verbal fluency, Trail Making Test, Rey Osterrieth complex figure recall, and the Cognitive Estimation Test). |
| Poyares et al., 2004 | Withdrawal | Of the 25 participants enrolled, 6 (24%) withdrew from the study due to difficulties with symptoms linked to BZD withdrawal. |
| Sato et al., 2020 | Mortality | The risk of cardiac death was significantly higher in patients taking BZD compared to those taking z-drugs (HR 1.45, 95% CI: 1.01-2.08; p=0.04). |
| Schenck and Mahowald, 1996 | Alcohol use disorder  Substance use disorder | Among patients with chronic insomnia, 8% had an alcohol use disorder in remission and 4% had a substance use disorder in remission. |
| Schneider-Helmert et al., 1988 | Mental performance  Physical performance | Self-reported mental performance was significantly lower after withdrawal from BZD use (p=0.05); however, physical performance was reported unchanged. |
| Schonmann et al., 2018 | Mortality  Addiction/ dependance/ substance abuse | Addiction- and dependance-related measures were significantly worse among z-drug users compared to BZD users, specifically after 10 years in long-term use, daily use, and excessive use.  However, rate of death was significantly higher in BZD users compared to z-drug users (43.3% and 37.1%, respectively; p<0.001). |
| Schredl et al., 1998 | Dream recall (cognition) | Memory, in the form of self-reported drug effects on dream recall, was assessed, finding the greatest effect to be on BZD users, followed by drug-free patients, and lastly, patients taking antidepressant. |
| Shapiro et al., 1995 | Alertness | The impacts of switching to a hypnotic with or without a gap in usage found that alertness was highest when the next medication was commenced immediately after the first. |
| Shash et al., 2016 | Dementia risk | An increased risk of dementia was reported in BZD users, compared to patients using a psychotropic medication (HR 1.10, 95% CI: 0.90-1.34). |
| Primary study:  Vignola et al., 2000;  Secondary study:  Bastien et al., 2003b | Cognition | On cognitive assessments, all patients with chronic insomnia (with or without BDZ use) performed worse than good sleepers on the Digit Span Forward test, assessing attention (p<0.01 for both comparisons), while those not taking BZD additionally performed worse on the Digit Span Backward test, assessing executive function (p<0.05).  No significant differences were found on tests of memory or other tests of attention (e.g., Digit Symbol Substitution) and executive function (e.g., Wisconsin Card Sorting Test). |
| Yen et al., 2015 | Substance abuse | Misuse of hypnotics was more likely in older adults using zolpidem compared to estazolam or flunitrazolam (OR 1.38, 95% CI: 0.60-3.18). |

* Conference abstract

**Abbreviations**: aOR: adjusted odds ratio; AVLT: Auditory Verbal Learning Test; BNT: Boston Naming Test; BZD: benzodiazepine; CBT: cognitive behavioural therapy; CI: confidence interval; DST: Digit span test; MMSE: Mini–mental state examination; MoCA: Montreal Cognitive Assessment; OR: odds ratio; STAI: State-Trait Anxiety Inventory; TMT-A: Trail Making Test, Part A; TMT-B: Trail Making Test, Part B.

Supplemental Table 12. Outcomes reported in studies included in the SLR assessing mental health outcomes (9 studies, 10 publications)

| Study | Measures assessed | Outcomes |
| --- | --- | --- |
| Bang et al., 2022 | BDI | A significantly higher score on the BDI was reported in the insomniacs using BZD group (mean 9.34 [SD 4.88]) compared to the control group (5.96 [5.03], p=0.02). |
| Bastien et al., 2003a | BDI  BAI | On both the BDI and BAI, all patients with insomnia (including those with and without BZD use) had significantly higher scores than the good sleepers group (p<0.01 across comparisons). |
| Chung et al., 2015 | Psychiatric disorders | Increased risk of several psychiatric disorders was reported to be significantly higher in insomnia patients with hypnotic use compared to individuals without insomnia or hypnotic use. The disorders included schizophrenia, bipolar disorder, depressive disorder, anxiety disorder, somatoform disorder, and substance-related disorder (p<0.001 across all comparisons).  Overall, similar findings were seen in the insomnia group without hypnotic use, when compared to individuals without insomnia or hypnotic use. |
| Declerck and Smits, 1999 | Depression symptoms  Anxiety symptoms | Patients who continue taking BZD did not show significantly different symptoms of depression or anxiety when compared to those who were switched to zolpidem. |
| Guo et al., 2021 | HAMD  HAMA | In patients with chronic insomnia categorized by presence or absence of cognitive impairment, measured in part by object cognitive assessments, both HAMA and HAMD scores were similar between groups (p=0.15 and p=0.32, respectively).  As well, when comparing patients based on BZD use (with or without use), there were no significant differences found between groups (p=0.77 on HAMD and p=081 on HAMA). |
| Lavigne et al., 2019 | Suicide risk | A comparison of incident suicide attempts among users of BZD and zolpidem did not find a significantly altered risk in one group over the other (BZD use: HR 1.33, 95% CI: 0.85-2.08). |
| Schenck and Mahowald, 1996 | Psychiatric disorders | Among patients with chronic insomnia, 80% had a past or current psychiatric disorder. |
| Song et al., 2020 | Depression symptoms  Anxiety symptoms | Self-reported symptoms of anxiety at 12 weeks were significantly greater in patients receiving JW-SZRD and lorazepam compared to lorazepam only (p<0.01), while no differences were reported on self-reported depressive symptoms. |
| Primary study:  Vignola et al., 2000;  Secondary study:  Bastien et al., 2003b | BDI  BAI | Worse symptoms of depression and anxiety were reported in patients with chronic insomnia (with and without BZD use) compared to good sleepers (p<0.01 for all comparisons). |

**Abbreviations**: BAI: Beck Anxiety Inventory; BDI: Beck Depression Inventory; BZD: benzodiazepine; CI: confidence interval; HAMA: Hamilton Anxiety Rating Scale; HAMD: Hamilton Depression Rating Scale; HR: hazard ratio; SD: standard deviation.

Supplemental Table . Outcomes reported in studies included in the SLR assessing QoL outcomes (2 studies, 2 publications)

| Study | Measures assessed | Outcomes |
| --- | --- | --- |
| Mah and Upshur, 2002 | Perceived risks and benefits | The perceived benefits of long-term BZD use were significantly higher in patients compared to physicians (3.84 and 2.84, respectively; p<0.001). The same was found for the perceived risks of long-term BZD use (3.63 in patients and 2.21 in physicians; p<0.001). |
| Song et al., 2020 | SF-36 | Scores on the SF-36 improved across all scales from baseline to week 12 in patients receiving JW-SZRD and lorazepam (treatment group) and those receiving lorazepam only (control group). |

Abbreviations: BZD: benzodiazepine; JW-SZRD: Jiawei Suanzaoren Decoction; QoL: quality of life; SF-36: 36-Item Short Form Survey.

Supplemental Figure 1. PRISMA flow diagram of the overall SLR process detailed for the two searches (Original SLR: 2012-2023, Updated SLR: 1987-2011)

Supplemental Figure 2. Overview of outcomes assessed by MA and NMA

* indicates NMA results for patients with chronic insomnia taking a BZD compared to the specified group.

Bold text indicates statistically significant results (p<0.05).

**Abbreviations**: BAI: Beck Anxiety Inventory; BDI: Beck Depression Inventory; BZD: benzodiazepine; ISI: Insomnia Severity Index; NE: not evaluated; PSQI: Pittsburg Sleep Quality Index; QoL: quality of life.
